# Mice Lacking the Circadian Modulators SHARP1 and SHARP2 Display Altered Sleep and Mixed State Endophenotypes of Psychiatric Disorders

**DOI:** 10.1371/journal.pone.0110310

**Published:** 2014-10-23

**Authors:** Paul C. Baier, Magdalena M. Brzózka, Ali Shahmoradi, Lisa Reinecke, Christina Kroos, Sven P. Wichert, Henrik Oster, Michael C. Wehr, Reshma Taneja, Johannes Hirrlinger, Moritz J. Rossner

**Affiliations:** 1 Department of Neurology, University of Kiel, Kiel, Germany; 2 Department of Clinical Neurophysiology, University of Göttingen, Göttingen, Germany; 3 Department of Psychiatry, Ludwig-Maximilian-University, Munich, Germany; 4 Research Group Gene Expression, Max Planck Institute of Experimental Medicine, Göttingen, Germany; 5 Circadian Rhythms Group, Max Planck Institute of Biophysical Chemistry, Göttingen, Germany; 6 Medical Department I, University of Lübeck, Lübeck, Germany; 7 Department of Physiology, National University of Singapore, Singapore, Singapore; 8 Carl-Ludwig Institute of Physiology, University of Leipzig, Leipzig, Germany; Hôpital du Sacré-Coeur de Montréal, Canada

## Abstract

Increasing evidence suggests that clock genes may be implicated in a spectrum of psychiatric diseases, including sleep and mood related disorders as well as schizophrenia. The bHLH transcription factors SHARP1/DEC2/BHLHE41 and SHARP2/DEC1/BHLHE40 are modulators of the circadian system and SHARP1/DEC2/BHLHE40 has been shown to regulate homeostatic sleep drive in humans. In this study, we characterized *Sharp1* and *Sharp2* double mutant mice (S1/2^-/-^) using online EEG recordings in living animals, behavioral assays and global gene expression profiling. EEG recordings revealed attenuated sleep/wake amplitudes and alterations of theta oscillations. Increased sleep in the dark phase is paralleled by reduced voluntary activity and cortical gene expression signatures reveal associations with psychiatric diseases. S1/2^-/-^ mice display alterations in novelty induced activity, anxiety and curiosity. Moreover, mutant mice exhibit impaired working memory and deficits in prepulse inhibition resembling symptoms of psychiatric diseases. Network modeling indicates a connection between neural plasticity and clock genes, particularly for *SHARP1* and *PER1*. Our findings support the hypothesis that abnormal sleep and certain (endo)phenotypes of psychiatric diseases may be caused by common mechanisms involving components of the molecular clock including SHARP1 and SHARP2.

## Introduction

The circadian system has been implicated in the control of alertness and clock genes have been associated with sleep and mood disorders, such as familial advance sleep phase syndrome (FASPS), depression, mania or bipolar disease (BD) [1]–[8], and therapeutic approaches modulating the circadian system (‘chronotherapy’) may be promising to improve treatment of psychiatric diseases [9], [10]. Sleep-wake behavior represents the most obvious behavioral output of the circadian system and nearly all psychiatric diseases including autism and schizophrenia (SZ) are characterized by irregular sleep-wake profiles [8], [11], [12]. In addition to the circadian control, homeostatic processes regulate vigilance states by increasing sleep drive and endurance [13]–[15].

Sleep serves a variety of vital functions. Prolonged wakefulness results in compensatory or rebound sleep, and disruption of normal sleep-wake cycles may contribute to psychiatric symptoms and inflammatory as well neurodegenerative processes [16], [17]; however, sleep deprivation has been shown to have short term beneficial effects in depressive patients [18], [19]. Reduced or disturbed sleep likely contributes to cognitive impairments and mood-related symptoms in psychiatric patients [8], [20]. Recent data support the notion that sleep is not only a distinct behavioral state but is rather characterized by defined cellular processes, as shown by monitoring sleep or wake associated cortical gene expression at a global scale [21], [22], and by progress in attributing sleep associated brain oscillations, i.e. in the theta or delta range, to functions in higher order neuronal plasticity [20], [23].

Sleep-wake behavior is altered in clock gene mutant mice, although the discrimination between circadian and homeostatic processes in mutants with a disturbed clock is difficult [24]–[28]. The mechanisms that link clock gene function with sleep-wake control and psychiatric diseases are just beginning to be explored [8]. For example, mice expressing a truncated version of the CLOCK protein (*Clock^Δ19^*) are characterized by hyperactivity, reduced sleep, lowered anxiety and depression-like behavior as well as impaired neuronal synchronizations [29], [30]. Moreover, mice lacking NPAS2, the functionally redundant paralog of CLOCK [31] which is, in contrast to CLOCK, prominently expressed in the forebrain [32], are also hyperactive, display reduced sleep and show altered sleep associated oscillations [25], [28].

SHARP1 (DEC2, BHLHE41) and SHARP2 (DEC1, BHLHE40) are negative regulators of CLOCK and NPAS2 and act as adaptation factors of the molecular clock [33]–[35]. Both genes are involved in the entrainment of the circadian system to altered external cues, yet the corresponding single and double null mutants are characterized by a slight period shift but do not display a disrupted circadian rhythm which indicates mainly functional core clock mechanisms [35], [36]. Period analysis, resetting to advanced and delayed light-dark (LD) cycles as well as nocturnal light pulses revealed gene dosage dependent functional redundancy between both genes [35] and genetic interactions with *Per1* and *Per2* [36], [37]. Moreover, SHARP1/DEC2 is involved in the regulation of sleep homeostasis in mice and humans [38]. A single point mutation in the C-terminal domain (P385R) found in a human family of ‘short’ sleepers reduces the transcriptional repressive activity of SHARP1. In a corresponding humanized mouse model, the duration of sleep is reduced and more fragmented compared to control animals, and the latter phenotype is strongly enhanced on a *Sharp1* null background [38]. With respect to sleep architecture, only a moderate increase of non-rapid eye movement (NREM) sleep during the early dark phase has been observed in *Sharp1* null mutants [38]. Therefore, the P385R mutant SHARP1 protein was considered to act in a dominant-negative fashion.

Given the mild sleep architecture phenotype in *Sharp1* null mutants and the functional redundancy of SHARP1 and -2 in the entrainment to external cues [35] it is possible that SHARP2 could at least partially compensate for the loss of SHARP1. Consequently, we analyzed sleep-wake behavior in *Sharp1* and *-2* double null mutant (S1/2^-/-^) mice. Our analysis revealed altered sleep architecture in S1/2^-/-^ mice with markedly attenuated light-to-dark amplitude of the different vigilance states. Moreover, daytime dependent changes in cortical gene expression and behavioral analyses revealed associations of SHARP1/2 function with endophenotypes of psychiatric diseases beyond the homeostatic control of sleep.

## Results

### Attenuated Sleep-Wake Amplitudes in S1/2^-/-^ mice

**Figure 1:**
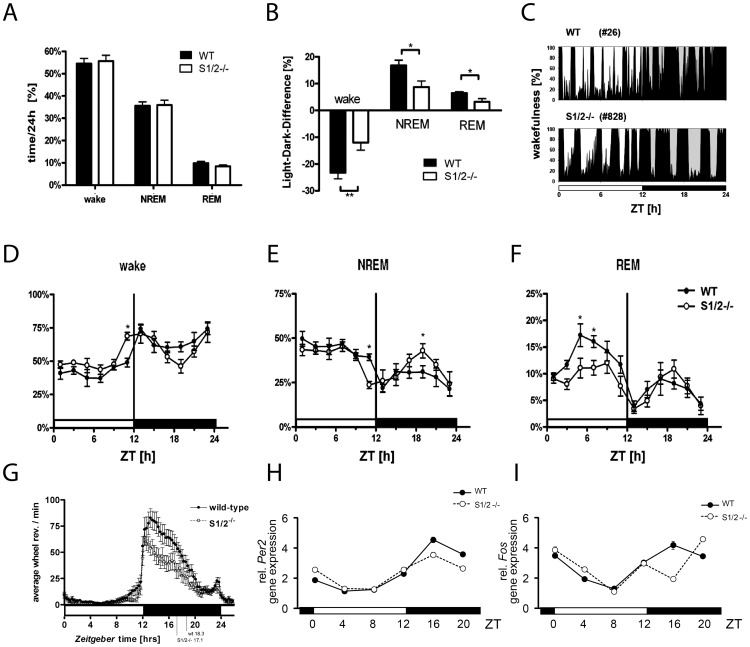
Attenuated sleep-wake amplitude and activity profiles in S1/2^-/-^ mice. A) Group means (± SEM) of the total time spend in different vigilance states over 24 h LD periods. The overall time of wake, NREM and REM sleep remained unaltered between genotypes. WT: n = 7, filled bars S1/2^-/-^: n = 8, empty bars. B) Group means of light-dark or amplitude differences for wake, NREM and REM sleep. S1/2^-/-^ animals showed a significantly reduced light-dark amplitude for all vigilance states compared to WT animals (E_genotype_ F_(1, 39)_ = 19.87, p<0.0001; E_vigilance state_ F_(2, 39)_ = 19.39, p<0.0001; I_genotype×vigilance state_ F_(2, 39)_ = 1.9, p = 0.16; Post hoc two-tailed T-test: **: p<0.01 *: p<0.05). WT: n = 7, filled bars S1/2^-/-^: n = 8, empty bars. C) 24 h sleep-wake distribution plotted for representative individual WT (#26) and S1/2^-/-^ (#828) mice with black areas given as relative amount of wakefulness obtained from 5 min bins. Note the relative difference in the amount of wakefulness during the light and dark episodes in the WT and the short periods of wakefulness in the light phase. In contrast, the S1/2^-/-^ mouse displayed broadened periods of sleep and wakefulness during the light and dark phases. D-F) Time course of vigilance states wakefulness (D), NREM (E) and REM sleep (F). Curves connect 2 h bin mean values (± SEM) expressed as percentage of recording time (E_time_: NREM: F_(11,120)_ = 9.74, p<0.0001; REM F_(11,120)_ = 9.98, p<0.0001; E_genotype_: NREM n.s.; REM F_(1,120)_ = 7.65, p<0.01 and I_genotype×time_: Wakefulness F_(11,120)_ = 2.06, p = 0.02; NREM: F_(11,120)_ = 1.82, p = 0.05; REM: F_(11,120)_ = 1.42, p =  0.17; * =  p<0.05 in two-tailed post hoc T-test. WT: n = 7, filled circles S1/2^-/-^: n = 8, empty circles. G) Diurnal wheel-running profiles depicted as accumulated activities of all recordings over a 5-day period plotted as 18 min bins. S1/2^-/-^ mice displayed a significantly altered activity profile in LD compared to wild-type (WT) mice (I_genotype×time_ F_(86,39040)_ = 1.92, p<0.0001) with reduced half maximal values of nocturnal activities at ZT 17.1 for S12^-/-^ mice compared to WT controls with ZT 18.3. Bonferroni posttest revealed significantly reduced activities between ZT13 and 18 (p_Bonf_<0.05). n = 12 each genotype. H-I) Daytime dependent gene expression analysis of the circadian marker gene *Per2* (H) and the activity-induced gene *Fos* (I) in the cortex. Daytime dependent cortical expression of the circadian marker gene *Per2* was not substantially altered in WT and S1/2^-/-^ mice (H). In contrast, the mRNA expression of the activity regulated marker gene *Fos* was significantly reduced in S1/2^-/-^ mice at ZT16 compared to WT (I). n = 3 per timepoint and genotype. Data were analyzed with 2-way ANOVA with Bonferroni posttest (p_Bonf_) and Mann-Whitney test (p_MW_) for pairwise comparisons. E, effect; I, interaction of factors.

Given the relatively mild sleep phenotype of *Sharp1* single mutants [38] and the functional redundancy of SHARP1 and -2 [35], we focussed on the analysis of *Sharp1* and *-2* double mutant mice (S1/2^-/-^). We performed EEG and EMG recordings on male mice to monitor for sleep-wake patterns over consecutive 24 h light-dark (LD) cycles. We determined the relative amounts of NREM (or slow-wave), REM (or paradoxical) sleep and wakefulness over 12 h light (L) and 12 h dark (D) periods and for the entire 24 h LD period. Total wakefulness, REM and NREM during 24 h of undisturbed sleep were similar in wild-type (WT) and S1/2^-/-^ animals (Figure 1A). However, there was a clear difference in the distribution of sleep and wakefulness during L and D. WT animals showed a substantial difference in the amount of wakefulness, NREM- and REM-sleep between L and D, whereas the sleep/wake amplitude was attenuated in S1/2^-/-^ animals (Figure 1B,C). Sleep-wake behavior was quantified as relative L-D differences, which were significantly reduced in S1/2^-/-^ animals for all three vigilance states (p<0.01 for wake, p<0.05 for REM and NREM sleep) (Figure 1B). The relative difference in the amount of wakefulness during the light and dark episodes is exemplified for individual WT and S1/2^-/-^ mice (Figure 1C). We plotted the cumulated EEG data in 2 h bins to increase temporal resolution (Figure 1D-F). NREM sleep was most prominently altered in the two hours preceding lights-off (zeitgeber time (ZT) 10-12; reduced NREM) and in the middle of the dark phase (ZT19-21; increased NREM; p<0.05; post-hoc test after 2-way ANOVA with significant genotype×time interaction p<0.05) (Figure 1E). REM sleep was reduced at ZT4-8 (p<0.05: post-hoc test after 2-way ANOVA with significant genotype p<0.01 and time p<0.0001 effects without significant genotype×time interaction) (Figure 1F).

We sleep deprived S1/2^-/-^ and control mice by gentle handling (ZT0-6 = L1, 97±1% efficient for both genotypes) to analyze homeostatic sleep drive (Figure S1A–C). The amount of NREM sleep after sleep deprivation (SD) revealed no significant differences in L2 (ZT7-12), at the beginning of D2 (ZT17) NREM sleep was increased in S1/2^-/-^ compared to WT mice (p<0.05) (Figure S1B). The relative amount of REM sleep, however, was significantly reduced in S1/2^-/-^ mice (p<0.05) compared to WT mice 2–4 h after the SD episode (ZT8-10) (Figure S1C). The analysis of slow-wave activity (SWA) or NREM delta power after SD revealed similar levels of rebound sleep in WT and S1/2^-/-^ mice compared to baseline values (Figure S2A). Nonetheless, we observed consistent but not significantly elevated SWA in S1/2^-/-^ mice at almost all time points independent of SD (Figure S2A). We also analyzed the REM sleep dominating synchronized oscillations in the 5–9 Hz range (theta) (Figure S2B-D). Theta peak frequency (TPF) was significantly higher at ZT7-12 in sleep deprived S1/2^-/-^ animals (TPF S1/2^-/-^: 7.60±0.12 Hz) compared to undisturbed (TPF WT 7.17±0.12 Hz; p<0.05) and sleep deprived WT mice (TPF WT: 7.22±0.03 Hz; p<0.01) (Figure S2B). Without SD, no spectral theta differences were observed in S1/2^-/-^ versus WT mice at ZT7-12 (Figure S2C). However, theta spectra were altered between sleep deprived S1/2^-/-^ and controls in the time period between ZT7-12 (Figure S2D; 2-way ANOVA, p = 0.0047). The increased TPF observed after SD in S1/2^-/-^ mice was not significantly different to the average values obtained for all sleep-deprived and naïve animals during the dark period and thus occurs in the normal physiological range (ZT13-18 and ZT19-24) (Figure S2B).

EEG analyses were complemented by assessing 24 h rest-activity profiles monitored with voluntary wheel running. In this assay, S1/2^-/-^ mice displayed normal entrainment to the LD cycle, but with a reduced wheel activity during D (Figure 1G), which correlates with the increased relative amount of sleep in D (Figure 1B). In L, we observed no significant differences in running wheel activities between the genotypes possibly due to photic masking of voluntary locomotor activity [39] (Figure 1G).

### Disturbed activity dependent gene expression

**Figure 2:**
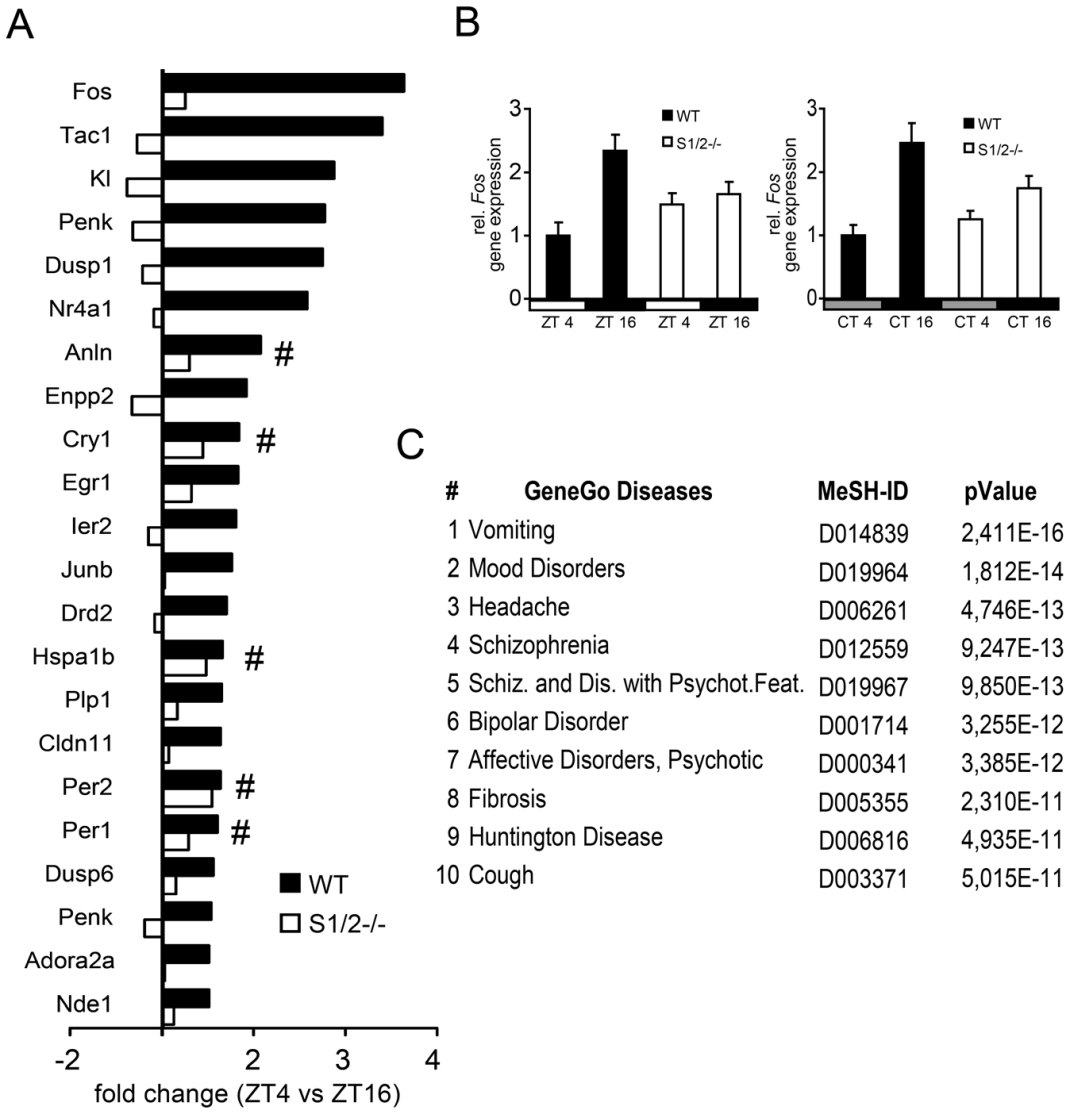
Cortical gene expression signatures correlate with psychiatric disease states. A) Depicted are fold-changes of differentially expressed genes at ZT4 versus ZT16 in the cortex of WT and S1/2^-/-^ mice detected by microarray analyses. In WT mice, several genes were upregulated with a fold-change of at least 1.5 at ZT16 (n = 22, *Penk* was detected by two independent probe sets) whereas this profile was strongly attenuated in S1/2^-/-^ mice with only 5 genes (marked with #) detected at the same cut-off (among those are 3 canonical clock genes *Cry1*, *Per1* and *Per2* as well as *Anln* and *Hspa1b*) that follow a normal although reduced ‘circadian’ profile. Note, that *Ttr* was for graphical reasons omitted because of its high fold-changes (Table S1). n = 2 per timepoint and genotype. B) Attenuated sleep-wake amplitude of *Fos* gene expression in the cortex of WT and S1/2^-/-^ mice in LD and DD. At ZT4 and ZT16 (where mice were kept under 12 h light and 12 dark conditions  =  LD; left panel) and at CT4 and CT16 (where mice were kept under constant darkness  =  DD; right panel) cortical *Fos* mRNA expression was analyzed with quantitative RT-PCR. n = 3 for each genotype. C) GeneGo enrichment analysis based on hyper-geometric statistics of the top 10 ranked disease associations of the cortical gene set (deregulated at ZT16 in S1/2^-/-^ cortex compared to WT). This analysis revealed highly significant correlations with neurological and particularly psychiatric disease classifications as indicated. Spelling of classifier 5 ‘Schizophrenia and Disorders with Psychotic Features’ is abbreviated as indicated. MeSH ID, unique Medical Subject Heading disease identifier.

Different vigilance states are characterized by specific cortical gene expression profiles [21]. To identify molecular correlates of the altered sleep-wake behavior and 24 h rest-activity profiles in S1/2^-/-^ mice, we first analyzed cortical gene expression of the circadian marker gene *Per2* and the activity-induced immediate-early gene (IEG) product *Fos* in 4 h bins over a complete 24 h cycle (Figure 1H,I). In line with previous observations [35] the circadian profile of *Per2* in the cortex was grossly normal, however, with a slightly but significantly reduced amplitude in S1/2^-/-^ versus WT mice (p<0.001, 2-way ANOVA) (Figure 1H). In contrast, the diurnal amplitude of cortical mRNA expression of the neuronal-activity marker *Fos* was preserved; but *Fos* transcription was strongly reduced at ZT16 in S1/2^-/-^ mice compared to WT animals (p<0.001, 2-way ANOVA; p<0.001 post-hoc test at ZT16) (Figure 1I) correlating with the elevated NREM sleep and reduced running wheel activity in D (Figure 1E,G). To obtain a more comprehensive insight into the changes in cortical gene expression in S1/2^-/-^ mice, we performed microarray analysis on cortical samples obtained from individual WT and S1/2^-/-^ mice harvested at two opposite time points of the LD cycle (ZT4 – wake phase; and ZT16 – sleep phase; Figure 2A; Table S1 and Table S2) where *Fos* expression differences were most prominent. This analysis revealed profound sleep-wake differences at the level of gene expression. Most prominently, the number of transcripts upregulated at ZT16 compared to ZT4 was substantially higher in WT (n = 22, corresponding to 21 genes with 2 probe sets detected for preproenkephalin *Penk*) than in S1/2^-/-^ mice (n = 5) (Table S1 and Table S2, see Table S3 for full gene name descriptions). In accordance with the quantitative (q)RT-PCR analysis (Figure 1H) and based on published results showing an intact circadian clock in S1/2^-/-^ mice [35], [36] among the five genes found to be significantly up-regulated in S1/2^-/-^ cortex at ZT16 were three canonical clock genes (*Per1, Per2, Cry1*) although at slightly reduced induction levels (Figure 2A and Table S1).

Wake-induced expression of transcripts encoding for neural plasticity genes (i.e. IEG transcription factors such as *Fos*, *Nr4a1*, *Egr1*, *Ier1* and *Junb* and those related to inter- and intracellular signalling such as *Tac1*, *Penk*, *Dusp1/6*, *Drd2*, *Adora2a*) was almost completely abolished in the S1/2^-/-^ mutants (Figure 2A and Table S1). Within this cluster of genes were also the oligodendrocyte/myelin markers *Enpp2*, *Plp1* and *Cldn11*. By qRT-PCR, we validated differential expression of selected genes in independent groups of mice in LD and DD (Figure 2B and Table S1). The analyses under constant darkness (DD) revealed a highly similar attenuated amplitude ruling out potential light masking effects.

Next, we used gene set enrichment analysis (GSEA) to identify cellular processes potentially altered in S1/2^-/-^ mice [40]. Similar to the gene-by-gene analysis, we detected the most profound differences when comparing WT and S1/2^-/-^ array data obtained at ZT16. Among the most significantly up-regulated gene sets in WT samples were several involved in cellular metabolism, such as components of the proteasome and ribosome as well as genes associated with oxidative phosphorylation (p-val<0.0001, FDR q-val<0.05) (Figure S3). Again, these molecular adaptations correlated with the attenuated sleep-wake/activity-rest amplitudes observed in S1/2^-/-^ animals. We also analyzed gene expression in cortical samples obtained from WT and S1/2^-/-^ mice after sleep deprivation. mRNA levels of two housekeeping genes, *Atp5b* and *Actb,* as well as *Per2* remained unaltered between WT and S1/2^-/-^ mutants (Figure S4B). In contrast, SD-induced down-regulation of *Per1*, *Fos* and *Egr1* gene expression observed in WT animals was markedly attenuated in S1/2^-/-^ mice correlating with genotype dependent REM sleep alterations at ZT10 (Figure S1C and S4B).

### Molecular signatures reveal associations with psychiatric disorders

**Figure 3:**
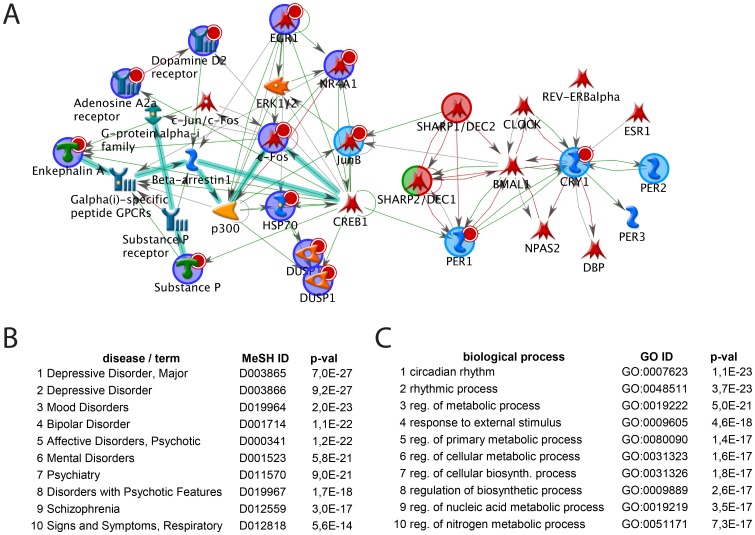
Unbiased network modeling links cortical signaling with clock components via SHARP1, BMAL1 and PER1. A) Depicted is the network model with the highest significance computed with all genes found to be differentially regulated in the cortex at ZT4 versus ZT16 (see. Figure 2A, Tables S1-S3 and Figure S9 for description of symbols) including SHARP1 and -2. The network connects 14 seed nodes depicted as blue circles (higher expression levels in WT are indicated by associated small red circles) extended by 13 interactors. SHARP1 and -2 are labeled by red circles; all nodes added by the algorithm are not underlined by colored circles. The structure depicts two major clusters and places the circadian regulators SHARP1 and SHARP2 as well as PER1 at central positions. The left cluster (n = 19 objects) is mainly comprised of cellular signaling components (enkephalin A, substance P both encoded by *Penk* and the GPCRs A2A and DRD2) and downstream effectors including negative regulators (DUSP1,6 and HSPs) as well as transcriptional mediators (e.g. FOS, EGR1, JUNB). The right cluster (n = 12) comprises central components of the molecular clock (e.g. the core clock transcription factors CLOCK, NPAS2 and BMAL1 as well as clock feedback regulators and modifiers including SHARP1 and -2, PER1 and -2, CRY1, DBP and NR1D1/Rev-ERBalpha). The extended network gene list was queried against the GeneGo database for enriched correlations with diseases (B) and biological processes (C). Among the ten most significant disease associations were nine mental or mood related disease classifications (B). Among the ten highest ranked biological processes were only circadian rhythm- (rank 1 and 2) and metabolism-associated (rank 3–8) processes (C). MeSH ID, unique Medical Subject Heading disease identifier; GO ID, unique identifier of the gene ontology biological process collection; p-values determined by hypergeometric tests.

In an unbiased approach, we applied software tools to detect potential associations of daytime-dependent cortical gene expression signatures (Table S1) in S1/2^-/-^ mice with particular disease classifiers [41]. Among the ten most significant matches were seven neurological medical subject heading (MeSH) classifiers and of those five were related to mood or psychotic disorders, suggesting a potential link between SHARP dysfunction and psychiatric diseases beyond alterations in sleep (Figure 2C). We further extended this analysis by applying pathway modeling algorithms to daytime-regulated genes including SHARP1 and -2 as seed nodes (Figure 3A). This approach aimed at detecting relationships between transcriptionally regulated as well as unregulated but functionally linked gene products to provide a more complete picture [42]. We used the most stringent shortest path algorithm that allows only 1-step indirect connections from curated literature, pathway and protein-interaction databases (see Methods). The corresponding network model predicted close functional interactions of 14 out of 23 gene products (64%) of the cortical gene expression signature (including SHARP1 and -2) and 15 connecting nodes (Figure 3A). Two distinct sub-clusters emerged from the network structure. One cluster connects components involved in neuronal signalling (enkephalin A, substance P, A2A and DRD2) and downstream effectors including negative regulators (DUSP1,6 and HSPs) as well as transcriptional mediators (e.g. FOS, EGR1, JUNB, NR4A1). The second cluster is comprised of central components of the molecular clock (e.g. the core clock transcription factors CLOCK, NPAS2 and BMAL1 as well as clock feedback regulators and modifiers including SHARP1 and -2, PER1 and -2, CRY1 and DBP and Rev-ERBalpha). Remarkably, only SHARP1, PER1 and BMAL1 were detected as connecting nodes between both clusters (Figure 3A). We queried the GeneGo databases with all components of this extended network to reveal associations with particular cellular process and diseases in an unbiased way. Among the top ten ranked disease associations were nine corresponding to psychiatric disorders including depressive, affective and psychotic MeSH classifiers (ranging from major depressive disorder [rank 1, p<10^−26^] to BD [rank 4, p<10^−23^] and SZ [rank 9, p<10^−16^]) (Figure 3B). Among the top ten ranked GO biological processes are two circadian rhythm related ones (circadian rhythm [rank 1, p<10^−22^] and rhythmic processes [rank 2, p<10^−22^]), and several that refer to central metabolic processes most likely reflecting the interaction between circadian clock function and cellular metabolic control [43]–[45] (Figure 3C).

### S1/2^-/-^ mice display endophenotypes of psychiatric disease

**Figure 4:**
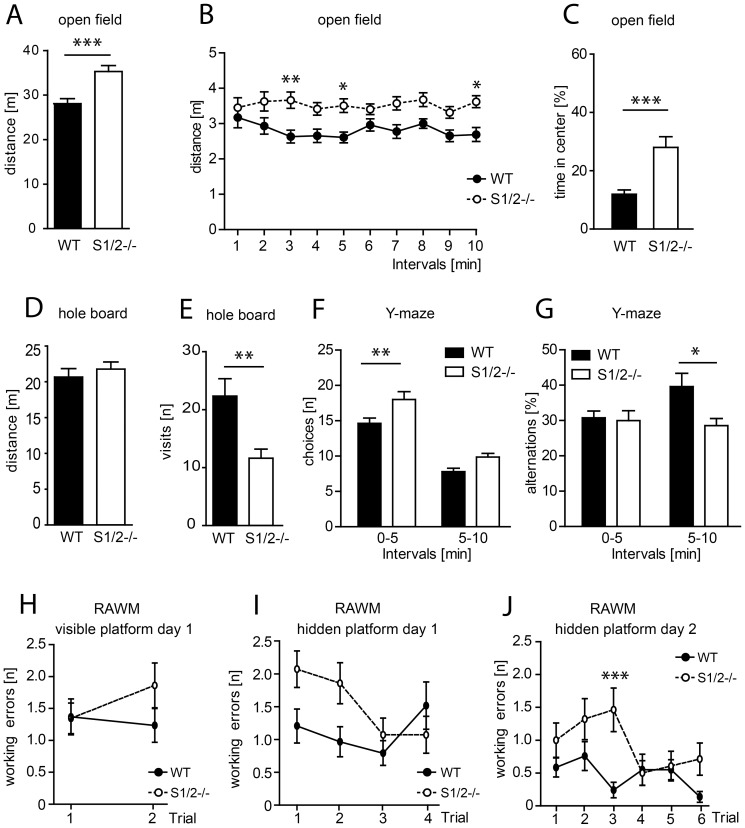
S1/2^-/-^ mice display novelty induced hyperactivity, decreased anxiety and exploratory behavior and working memory disturbances. A–C) Open field test performed in a novel, unfamiliar test arena. WT: n = 24, S1/2^-/-^: n = 26. A) Novelty-induced hyperactivity in S1/2^-/-^ mice as assessed by moving distance in the open field (p_MW_ = 0.0006). B) Analysis in 1 min bins yielded a significant E_genotype_ (F_(1,48)_ = 16.46; p = 0.0002). Moreover, Bonferroni posttest revealed the strongest difference between the genotypes in interval 3, 5 and 10 (p_Bonf_<0.01, p_Bonf_<0.05 and p_Bonf_<0.05, respectively). C) Mutants spent more time in the center (p_MW_ = 0.0004) of the test arena indicating reduced anxiety when compared to controls. D-E) Hole board test performed with a subsequent modification of the open field setup by floor insert with holes. WT: n = 24, S1/2^-/-^: n = 26. D) S1/2^-/-^ mice displayed no alterations in the overall activity measured as total distance travelled. E) S1/2^-/-^ mice performed less nose pokes into holes (p_MW_ = 0.0014) indicating decreased curiosity-related behavior compared to WT. F-G) Y-maze test. WT: n = 23, S1/2^-/-^: n = 20. F) S1/2^-/-^ mice showed increased activity in Y-maze test (E_genotype_ F_(1,41)_ = 10.98; p = 0.0019) most evident in interval 0-5 min (p_Bonf_<0.01). G) Mutant mice performed less alterations in Y-maze than control animals (E_genotype_ F_(1,41)_ = 4.86; p = 0.0331) and p_Bonf_<0.05 for interval 5–10 min. H-J) S1/2^-/-^ mice display impairment of working memory in the radial arm water maze (RAWM). WT: n = 29, S1/2^-/-^: n = 28. H) In the visible platform task, performance was similar in both genotypes (E_genotype_ F_(1,55)_ = 0.65; p = 0.4236). I-J) S1/2^-/-^ mice showed increased number of working errors searching for a hidden platform on the first (I) (E_genotype_ F_(1,55)_ = 3.93; p = 0.0524; I_genotype×time_ F_(3,165)_ = 2.68; p = 0.0486) and the second (J) day of experiment (E_genotype_ F_(1,55)_ = 9.05; p = 0.0044) and I_genotype×time_ F_(5,275)_ = 2.34; p = 0.0422). Bonferroni posttest revealed significant difference during the 3^rd^ trial of the second day (p_Bonf_<0.001). WT, black bars/circles. S1/2^-/-^, white bars/circles. Data were analyzed with 2-way ANOVA with Bonferroni posttest (p_Bonf_) and Mann-Whitney test (p_MW_) for pairwise comparisons. ***: p<0.001; **: p<0.01; *: p<0.05. E, effect; I, interaction of factors.

**Figure 5:**
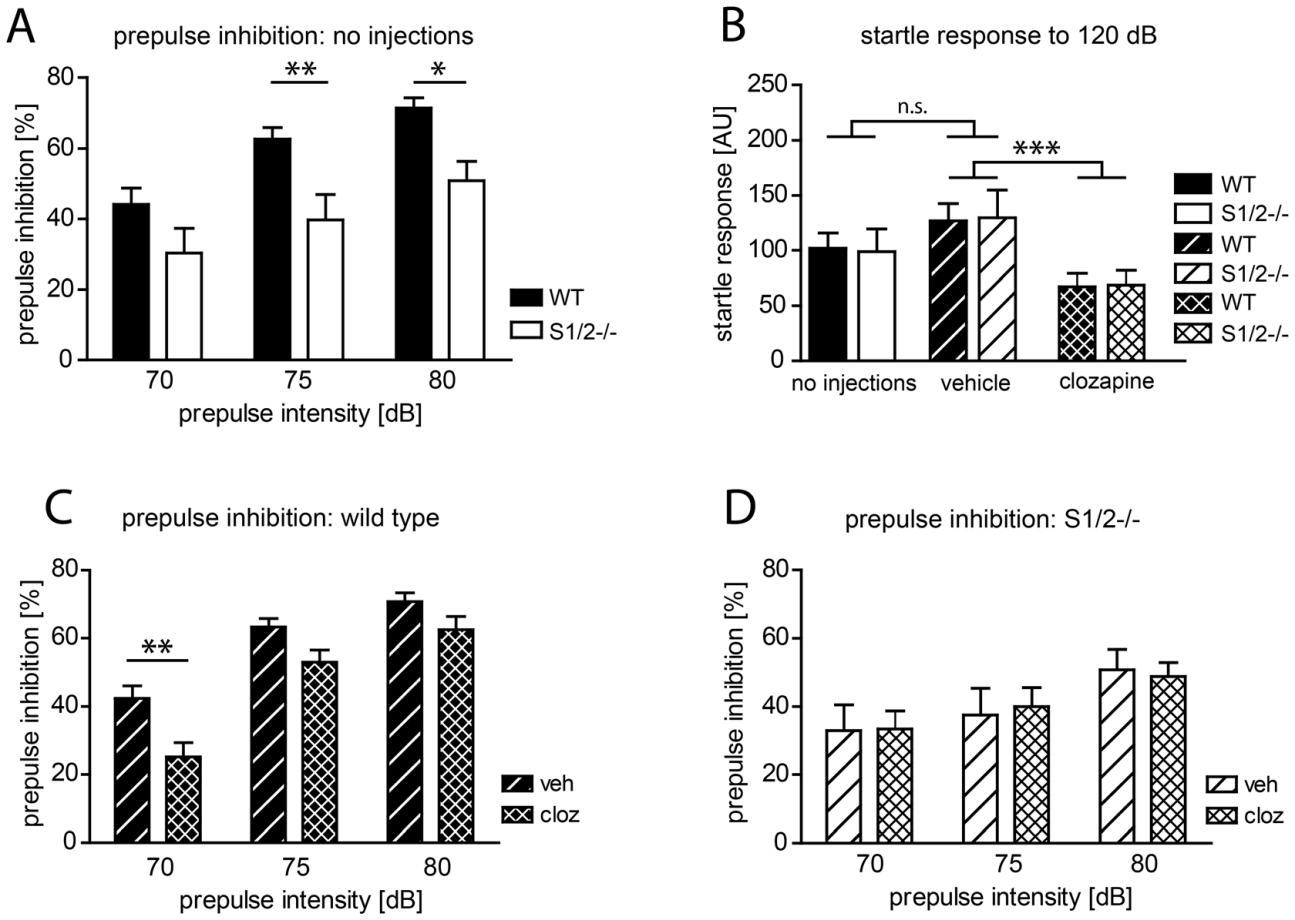
S1/2^-/-^ mice show alterations of prepulse inhibition (PPI) which are resistant to Clozapine treatment. A) S1/2^-/-^ mice display impairment of PPI (E_genotype_ F_(1,43)_ = 7.99; p = 0.0071). Bonferroni posttest revealed significant difference in prepulse 75 und 80 dB (p_Bonf_<0.01 and p_Bonf_<0.05, respectively). WT: n = 24, S1/2^-/-^: n = 21. B) Startle response was similar in S1/2^-/-^ mice and WT controls (E_genotype_ F_(1,88)_ = 0.00; p = 0.9958) and not influenced significantly by vehicle injections (E_treatment_ F_(1,88)_ = 2.18; p = 0.1434). Acute clozapine treatment (3 mg/kg) reduced startle in both genotypes to similar extend (E_treatment_ F_(1,82)_ = 11.83; p = 0.0009 and E_genotype_ F_(1,82)_ = 0.01; p = 0.9030). ‘No injections’ and ‘vehicle’ groups: WT: n = 25, S1/2^-/-^: n = 21; clozapine: WT: n = 20, S1/2^-/-^: n = 20. C) Acute treatment with clozapine (cloz; 3 mg/kg; n = 20) reduced PPI in WT mice when compared to vehicle (veh; n = 24) treated WT animals (E_treatment_ F_(1,42)_ = 10.33; p = 0.0025). Bonferroni posttest confirmed significant difference when prepulse 70 dB was applied (p_Bonf_<0.01). D) Acute treatment with clozapine (cloz; 3 mg/kg) did not influence PPI in S1/2^-/-^ mice (n = 20) when compared to vehicle injected mutants (n = 21) (E_treatment_ F_(1,39)_ = 0.00; p = 0.9716). Data were analyzed with 2-way ANOVA and Bonferroni posttest (p_Bonf_). ***: p<0.001; **: p<0.01; *: p<0.05. E, effect.

EEG recordings revealed a role of SHARP1/2 in the homeostatic control of sleep and unsupervised analysis of cortical gene expression profiles provided a possible link to psychiatric diseases, particularly mood and psychotic disorders. Therefore, we analyzed S1/2^-/-^ mice to assess behavioral aspects that may be relevant in the context of psychiatric diseases: including basic behavior (motivation, exploratory, curiosity and anxiety), working memory performance and sensorimotor gating (Figure 4–5 and Figure S5–S8). In the open field test, we observed a highly significant novelty-induced hyperactivity in an unfamiliar environment (p_MW_ (p value for Mann-Whitney test) <0.0006; Figure 4A), persistent during the entire test (effect of genotype p = 0.0002; 2-way ANOVA, Figure 4B) and most prominent in interval 3, 5 and 10 (p_Bonf_ (p values for Bonferroni post test) <0.01, <0.05 and <0.05, respectively;). S1/2^-/-^ mice spent more time in the center (p_MW_<0.0004) of the test arena than controls possibly indicating reduced anxiety or risk-taking behavior (Figure 4C). Elevated plus maze (Figure S5A) and light-dark preference test (Figure S5B), however, did not reveal alterations of anxiety-related behavior. S1/2^-/-^ mice spent similar time in closed arms in elevated plus maze (p_MW_ = 0.8693) and in the dark compartment during light-dark preference test when compared with WT controls (p_MW_ = 0.1917). On the subsequent day after the open field test, the same test arena was equipped with a ‘hole-board’ to monitor nose pokes as an indicator of exploratory drive, curiosity-related behavior. Whereas the total travelled distance did not differ between the genotypes under more familiar conditions (Figure 4D), the number of nose pokes (Figure 4E) was significantly reduced in S1/2^-/-^ versus WT mice (p_MW_ = 0.0014). However, motivational behavior as assessed in tail suspension test (p_MW_ = 0.9456; Figure S5C) and in sucrose preference test (p = 0.1719; 2-way ANOVA, Figure S5D) was unaltered.

As impairment of working memory is one of the core symptoms of SZ and BD [46]–[48], we addressed working memory performance of S1/2^-/-^ mice using the Y-maze test. S1/2^-/-^ animals displayed an increased activity in this novel environment performing more arm choices (p = 0.0022; 2-way ANOVA, Figure 4F), mainly during the first 5 min (p_Bonf_<0.01). Mutants showed less alterations in Y-maze than controls (p = 0.0331; 2-way ANOVA, Figure 4G) with the lower performance during interval 5–10 min (p_Bonf_<0.05). We also observed working memory deficits in a radial arm water maze (Figure 4H–J). In the visible platform task, performance was similar in both genotypes (p = 0.4236; 2-way ANOVA, Figure 4H) but S1/2^-/-^ mice displayed more working errors searching for a hidden platform on the first (p = 0.0524; 2-way ANOVA, interaction genotype×time p = 0.0486; Figure 4I) and on the second day of the experiment (effect of genotype p = 0.0044 and genotype × time p = 0.0422; 2-way ANOVA, Figure 4J) with the most prominent difference during trial 3 (p_Bonf_<0.001; Figure 4J).

To assess sensorimotor gating as an additional endophenotype of several psychiatric diseases [49]–[51], S1/2^-/-^ mice and WT controls were tested in a prepulse inhibition (PPI) test under normal conditions and after treatment with clozapine. We performed the tests on 3 consecutive days with naïve animals (day 1), injected with vehicle (day 2) and after acute clozapine treatment (3mg/kg; day 3).

To exclude possible effects of multiple testing on PPI, we first exposed naïve wild type animals (age-matched male C57Bl/6) on three consecutive days to PPI (Figure S6). We did not observe effects of multiple testing on PPI (p = 0.1539; 2-way ANOVA; Figure S6A) nor on startle response (p = 0.9724, 1-way ANOVA; Figure S6B).

Naïve S1/2^-/-^ mice displayed pronounced impairment of PPI (p = 0.0071; Figure 5A) with most significant difference at prepulse 75 and 80 dB (p_Bonf_<0.01 and p_Bonf_<0.05, respectively). Startle response (Figure 5B) was similar in S1/2^-/-^ mice and WT controls (p = 0.9958; 2-way ANOVA) and was not influenced significantly by vehicle injections (p = 0.1434; 2-way ANOVA).

Acute clozapine treatment (3 mg/kg) reduced startle response in both genotypes to similar extend when compared with vehicle injections (p = 0.0009; 2-way ANOVA, Figure 5B). Clozapine diminished PPI in WT mice when compared to vehicle treated animals (p = 0.0025; 2-way ANOVA, Figure 5C). Bonferroni posttest yielded significant difference at 70 dB prepulse (p_Bonf_<0.01). Clozapine had no effects on PPI in S1/2^-/-^ mice (p = 0.9716; 2-way ANOVA, Figure 5D).

S1/2^-/-^ and control mice showed habituation to 120 dB pulse during PPI assessment (naïve: p<0.0001; Figure S7A; vehicle: p = 0.0006; Figure S7B; clozapine: p = 0.0197; Figure S7C; 2-way ANOVA) which was comparable between the genotypes in all treatment groups (naïve: p = 0.4766; vehicle: p = 0.6974; clozapine: p = 0.6008; 2-way ANOVA).

We also assessed effects of vehicle and clozapine (3 mg/kg) injections in the open field (Figure S8). Vehicle injection reduced hyperactivity in S1/2^-/-^ mutants and increased anxiety in WT and to a lesser extend also in S1/2^-/-^ mutants (p_MW_ = 0.2380, Figure S8A and p_MW_ = 0.2250, Figure S8B). Clozapine treatment dramatically reduced the overall activity (p<0.0001; Figure S8A) and time spent in the center (p = 0.0009; Figure S8B) in both genotypes. However, clozapine effects were stronger on S1/2^-/-^ mice regarding distance (interaction genotype × treatment p = 0.0296, 2-way ANOVA) and time in center (interaction genotype × treatment p = 0.0665, 2-way ANOVA).

## Discussion

### Altered sleep architecture in S1/2^-/-^ mice

In this study, we have analyzed the sleep architecture, daytime-dependent gene expression in the cortex and behavior in SHARP1 and SHARP2 double deficient (S1/2^-/-^) mice. Our data show that sleep-wake profiles, responses of REM sleep and theta oscillations to sleep deprivation, running wheel behavior and activity-related cortical gene expression are altered in S1/2^-/-^ animals, although the 24 h cumulated total amount of sleep and wakefulness and overall circadian rhythmicity remain unaltered. The alteration of sleep architecture in S1/2^-/-^ mutants, however, does not phenocopy mice that express the human SHARP1/DEC2(P385R) protein which display a reduction in total sleep time [38]. This implies that the P385R mutant SHARP1 protein does not act in a ‘simple’ dominant-negative fashion interfering with SHARP1/2 repressor functions [38], i.e. by forming heterotypic homo- or heterodimers with ‘wild-type’ proteins. Since SHARP1 and SHARP2 can homo- and heterodimerize [52] and function in a context dependent fashion either as repressors and co-activators of CLOCK, NPAS2 as well as other transcriptional regulators [35], it might be possible that the mutant SHARP1/DEC2(P385R) protein could be mechanistically specific e.g. by only affecting discrete repressive functions. This hypothesis should be addressed experimentally and might be helpful in understanding the molecular mechanisms that cause distinct sleep phenotypes in S1/2^-/-^ mice versus those expressing the SHARP1/DEC2(P385R) mutant protein. In addition, S1/2^-/-^ mice display alterations e.g. in REM sleep that have not been observed in SHARP1 single mutants [38]. Moreover, we found that REM sleep associated theta oscillations were altered in S1/2^-/-^ mice upon sleep deprivation. Thus, loss of SHARP function causes an altered adaptability of environmental stressors at the level of neuronal synchronizations which may be of relevance for psychiatric disease related behavior such as anxiety [30], [53].

### SHARP1/2 mutants display ‘mixed-state’ endophenotypes of psychiatric diseases

In a novel environment, S1/2^-/-^ mice display locomotor hyperactivity and diminished anxiety comparable to mania-like behavior. Diminished anxiety can be interpreted as increased risk taking [54], one of symptoms of BD [55]. However, alterations in other anxiety tests were absent; possibly because different anxiety tests monitor distinct types of emotional behavior as suggested previously [56], [57]. Contrasting anxiety phenotypes have also been found in mice haploinsufficient for the SZ/BD risk gene CACNA1c [58], [59] and in mice heterozygous for the BD associated gene *Ank3* showing altered behavior in elevated plus maze and light-dark preference but not in the open field test [60]. However, no hyperactivity was detected in S1/2^-/-^ animals in a more intimate environment resembling the phenotype of mice with a dopamine transporter knockdown where mania-like exploratory behavior is present in novel but diminished in a familiar environment [61]. In S1/2^-/-^ mice, specific exploration measured by nose pokes was diminished resembling more depression-like symptoms. Similarly, when monitored for the 24 h voluntary locomotor activity profile in home cage, S1/2^-/-^ mice displayed hypoactivity during the dark phase (D) which correlates with the observed relative increase of sleep in D. The locomotor hypoactivity, reduced exploratory drive and increased sleepiness in the active phase in S1/2^-/-^ mutants are more reminiscent of a depression-like state. Mixed-state or paradoxical phenotypes of mania- and depression-like behavior have previously been observed when inactivating CLOCK selectively in the hypothalamus [62]. Mouse mutants lacking functional CLOCK or NPAS2 show locomotor hyperactivity and reduced sleep in D [28], [29], which has been interpreted as mania-like behavior in CLOCK deficient mice [29]. Therefore, SHARP1/2 mutant mice meet selected ‘mixed-state’ criteria for face validity towards BD similarly to *Clock* mutants [63]. Nonetheless, it is still unclear which of the mixed-state phenotypes are a direct or indirect consequence of loss of SHARP1/2 functions. It is possible that feedback mechanisms operate at the molecular and behavioral level that cause mutual relationships e.g. between sleep and affective phenotypes seen in S1/2^-/-^ mice.

### Altered endophenotypes at the circuitry level

The spectrum of endophenotypes in S1/2^-/-^ mice with relevance for psychiatric diseases is further expanded by the working memory impairments and PPI deficits. Working memory deficits are prominent in SZ and BD [46]–[48] and corresponding animal models [64]–[68]. In concordance with published data, acute clozapine treatment reduced PPI in WT [67] but had no further effect on the low PPI levels in S1/2^-/-^ mice. Interestingly, clozapine is also not effective in more than 50% of treatment resistant schizophrenic patients [69]. Although clozapine had differential effects on PPI, it reduced to a similar extend startle amplitude in both S1/2^-/-^ mutant and control mice which has been described in mice [67], [68] and human subjects [70]; however, showing stronger sedative effects on S1/2^-/-^ mutants. Resistance of S1/2^-/-^ mice towards clozapine in PPI may be partially related to the blunted DRD2 expression in these mutants [71], [72], although other mechanisms might also be involved given the broad pharmacological profile of clozapine. Therefore, S1/2^-/-^ mice may be a valuable model to test novel compounds or therapies to overcome clozapine resistance.

### Cortical gene expression profiling reveals associations with psychiatric diseases

We applied unbiased bioinformatic algorithms to identify biological process and disease associations with gene sets obtained from cortical gene expression profiling in WT and S1/2^-/-^ mice [41], [42]. These analyses use curator-indexed literature databases and do not provide direct experimental evidence. Nonetheless, these analyses identified several psychiatric diseases (particularly mood- and psychotic disorders) correlating with SHARP1/2 dysfunction-associated cortical gene expression. To substantiate these findings, we scanned the mouse phenotype database provided by the Jax labs (www.informatics.jax.org/phenotypes.shtml) and found that *Drd2* and *Adora2a* mouse mutants were associated with ‘hypoactivity’ (mouse phenotype ID MP:0001402) [73], [74] and display altered behavioral adaptability to psychostimulants [75], [76]. Polymorphisms in *ADORA2A* may modulate psychomotor vigilance and sleep EEG [76] and it has been previously noted that *DRD2* variants are associated with mood disorders [77]. In addition, DRD2 represents an interesting pharmacological target for the treatment of psychosis [78] and ADORA2A has been suggested as a target for the treatment of psychiatric diseases [79]. Moreover, we detected attenuated gene expression of three myelin genes (*Enpp2*, *Plp1*, *Cldn11*) in the cortex of S1/2^-/-^ mice. This finding provides an additional link with psychiatric diseases since reduced expression of oligodendrocyte/myelin markers is among the most replicated molecular observations in psychiatric diseases including SZ and major depression, although an underlying mechanistic concept for these observations is still missing [80]–[82].

### Disintegration of activity-dependent and circadian processes

Among the few upregulated genes in the cortex of S1/2^-/-^ mice at ZT16 were three canonical circadian factors (*Per1*, *Per2* and *Cry1*) indicating a functional clock in agreement with previous observations [35], [83]. The sleep-wake amplitude in expression of these circadian genes, however, was attenuated, although not completely abolished, as compared to that of plasticity-related genes. It has been noted before that the expression of clock regulated genes in the cortex is not strictly controlled by circadian mechanisms only [21] which may explain the partially reduced amplitude under LD and DD conditions in S1/2^-/-^ mice. We also detected the genes *Penk* and *Tac* encoding the neuropeptides preproenkephalin and tachykinin as deregulated in the cortex of S1/2^-/-^ mice. These hormones have been associated with anxiety, analgesic effects and altered stress responses [84], [85]. It is therefore possible that these and potentially other neuroendocrine factors may have caused or modulated some of the behavioral alterations observed in S1/2^-/-^ mice.

Sleep-wake behavior is regulated by circadian and homeostatic processes [13]–[15]. Therefore, the attenuated running wheel profile of S1/2^-/-^ mice in L, which is paralleled by increased NREM sleep levels and reduced activity-regulated gene expression, could be driven by disturbance of a thus far unknown homeostatic process. In consequence, an uncoupling of homeostatic and circadian processes could explain the molecular and behavioral alterations seen in S1/2^-/-^ mice. The identification of the postulated homeostatic process(es) altered upon loss of SHARP1 and -2 could help to better understand the link between the disturbed sleep architecture and the molecular and behavioral consequences.

Network modelling of all deregulated genes including SHARP1 and SHARP2 revealed a bipartite assembly of activity/plasticity genes and circadian factors that were connected by SHARP1 and PER1. It may thus be possible that particularly SHARP1 and also PER1 may be key regulators integrating plasticity-related as well as circadian and homeostatic processes in the cortex. As shown in this study, the loss of SHARP function results in a disintegration of both processes consequently leading to psychiatric (endo)phenotypes. Recent observations made with *Sharp1/2* and *Per1* triple null mutant mice, however, revealed a genetic interaction of these factors in the regulation of circadian locomotor activity [37]. In light of our findings presented in this study, it might thus be interesting to characterize sleep and behavior also in *Sharp1/2* and *Per1* triple mutants.

In summary, our findings support the hypothesis that abnormal sleep and certain (endo)phenotypes of psychiatric diseases may be caused by common mechanisms involving components of the molecular clock including SHARP1 and SHARP2. Moreover, genetically defined mouse models of circadian genes with distinct (endo)phenotype profiles, such as S1/2^-/-^ mice, may be useful for pre-clinical treatment trials in the context of psychiatric diseases including sleep and mood disorders.

## Materials and Methods

### Animal experiments

All animal experiments were conducted in accordance with NIH principles of laboratory animal care and were approved by the Government of Lower Saxony, Germany. The experiments were performed with cohorts of adult male mice aged 3–5 months, respectively. Cohorts of mice within experiments were age matched (±2 weeks). Parental single heterozygous mice were independently backcrossed to C57Bl/6J for more than ten generations as described previously [35]. Wild-type (WT) and *Sharp1* and *-2* double mutant mice (S1/2^-/-^) were obtained from double heterozygous breeding pairs [35], [86]. All experimental animals were group housed in the same ventilated sound-attenuated rooms under a 12 h light/12 h dark schedule at an ambient temperature of 21°C with food and water available *ad libitum*. All experiments were performed blinded to genotypes.

### Surgical implantation procedures

Surgery was performed under deep intraperitoneal anaesthesia with Ketamine/Xylazine (100 mg/kg; 10 mg/kg). Two stainless steel screws (diameter 0.7 mm; Plastics One) were implanted epidurally in the skull over the right parietal cortex (1.7 mm lateral to midline, 1.0 mm anterior to lambda) and the left frontal cortex (1.7 mm lateral to midline and 1.5 mm anterior to bregma) to derive the electroencephalogram (EEG). A third screw over the left hemisphere served as anchor screw. For electromyographic (EMG) recordings two stainless steel wires were inserted into the neck muscles bilaterally. All electrodes were connected to a mouse-adapted socket (Plastics One), which was fixed with dental acrylic cement.

### EEG Recordings

After surgery the animals were allowed to recover for at least 2 weeks before data acquisition. To habituate to recording conditions animals were connected to the recording lead attached to a swivel contact at least 4 days before the start of experiments. Recordings were performed on three consecutive 24 h periods starting at lights-on. Day one was not analyzed, day two served as baseline recordings and on day three animals were sleep deprived by gentle handling. EEG and EMG signals of four animals (two of each genotype) were recorded simultaneously to equally distribute environmental disturbances. EEG and EMG signals were amplified, filtered, analog-digital-converted at 256 Hz and stored on a computer hard disk (Sleep Sign Acquisition, Kissei Comtec). Three vigilance states, wakefulness (W), non rapid eye movement sleep (NREM) and rapid eye movement sleep (REM), were determined offline (Sleep Sign Analysis, Kissei Comtec) in epochs of 4 s by visual assessment of EEG- and EMG-recordings. Epochs containing more than one vigilance state were scored as the prevailing one and epochs containing recording artefacts were omitted from subsequent spectral analysis. The amount of each vigilance state was expressed as percentage of recording time.

### Spectral analysis and measurement of slow-wave-activity

For each 4 s epoch scored as NREM or REM sleep, EEG was subjected to a fast Fourier Transformation (FFT) analysis, yielding power spectra between 0.5 and 20 Hz with a 0.5 Hz resolution (Sleep Sign Analysis, Kissei Comtec). Slow wave activity (SWA) in NREM sleep was calculated as the mean power over the frequency range between 0.5 and 4.0 Hz. All SWA measures were expressed as percentage of the individual mean SWA over the last 900 NREM epochs in the baseline light period to correct for individual differences in the absolute power.

### Spectral analysis and determination of theta peak frequency

EEG spectral profiles of REM sleep are dominated by frequencies in the Theta band range. To determine the prevailing frequency in REM sleep, the distribution of peak frequency was calculated. Means of FFT spectra of artefact-free 4 s epochs were calculated over two-hour intervals and the frequency of the maximal power in the range between 5 and 10 Hz determined for these mean frequency distributions.

### Running wheel analysis of activity profiles

Activity data were recorded and evaluated using the ClockLab data collection and analysis software (Actimetrics) as described previously [35]. To obtain a full activity profile, 24 h accumulated activities of running wheel recordings over a 5 day period were analyzed in 18 min bins.

### Tissue Sampling and gene expression analysis

Cortical tissue (bregma 0 to −2 mm) was isolated using a ‘rodent brain matrix’ 1 mm coronal slicer (ASI Instruments, Warren, MI) from adult WT and S1/2^-/-^ mice harvested at ZT0, ZT4, ZT8, ZT12, ZT16, ZT20 and independent cohorts analyzed at ZT4, ZT16, CT4 and CT16 (n = 3 for each genotype and time point). Animals were kept 24 h under DD (12 h dark, 12 h dark) before being sacrificed under dim red light for the analysis of all CT time points. To analyze gene expression in response to sleep deprivation, cortical samples were collected as described above with a rodent brain matrix and pooled (n = 4 each time point and genotype). RNA was prepared according to the manufacturer’s protocols using RNeasy colums (Qiagen, Hilden, Germany) and analyzed for integrity using the Bioanalyzer (Agilent Technologies). The minimal RNA-integrity (RIN) value threshold was 8.5. For microarray analysis, one-round RNA amplification, labeling and hybridization were essentially performed as previously described [87]. Microarrays were scanned and pre-processed according to standard protocols given by the manufacturer (Affymetrix). Array data were analyzed using either R-scripts (www.bioconductor.org) or the Genomics Suite (Partek). Differential gene expression over time was determined using ANOVA and for genotype comparisons by applying moderate T-statistics (using the corresponding bioconductor package). Selection cut-offs were set to fold-changes>1.5 and corrected p-values <0.05. Gene set enrichment analysis (GSEA) of *a priori* defined sets of functionally grouped genes was performed using the GSEA software package downloaded from www.broadinstitute.org/gsea and implemented locally. Analyses were performed with default parameters (permutations set to 1000) and gene sets available in the molecular signature database (MSigDB v3.0) as described previously [87].

Quantitative PCR was performed with an ABI PRISM 7700 detection system (Perkin Elmer), essentially as described [88]. Primers directed against mouse transcripts (Table S4) were designed online at the Roche assay design center (www.roche-applied-science.com/sis/rtpcr/upl/) and used in SYBRgreen assays.

### Gene Ontology and network analysis

Gene Ontology (GO) analysis was performed with Genomics Suite (Partek) and MetaCore (GeneGo) using categories provided by the GO consortium (www.geneontology.org). Gene-disease association and network modelling was performed with MetaCore (GeneGo) using manually curated disease databases compiled from RefSeq annotations (http://www.ncbi.nlm.nih.gov/refseq/rsg/) and literature minings listed as interlinked pubmed entries with each gene in the corresponding disease database as implemented in the software (www.genego.com). Dijkstra’s shortest path algorithm with a maximum of steps set to 1 was applied for network modeling and hypergeometric tests for GO enrichment and gene/disease associations as implemented in the software.

### Behavioral analysis

Mice were tested for free running activity (in home cage), in open field, hole board, elevated plus maze, light-dark preference, tail suspension test, hot plate, Y-maze, radial arm water maze and prepulse inhibition. Behavioral tests monitoring for novelty induced activity, anxiety and curiosity (open filed and hole board), anxiety (elevated plus maze and light-dark preference), pain sensitivity (hot plate), escape motivation and/or depression (tail suspension) and sensorimotor gating (prepulse inhibition test) were essentially performed as described previously [64] Experiments were performed between ZT2-ZT6 during light phase or between ZT 14-18 in the wake-phase and under dim red light where indicated.

### Light-dark preference

The light dark preference test was conducted in a box consisting of compartments of the same size: one with black walls (dark) connected by a door with light compartment build of transparent Plexiglas. Mice were placed in the transparent compartment facing the wall and left undisturbed. Latency to enter the dark, the time spent in the dark box and crossings between two compartments were scored for 5 min.

### Y-maze

Y-maze was performed using a custom made Y-shaped runway. Mice were put into maze facing the wall and allowed to explore undisturbed the maze for 10 min. The experiment was video recorded and analyzed offline. The number of arm choices (as a measure of activity) and the percent of alterations (choices of a “novel” arm, different than chosen before as a measure of working memory) were scored. The apparatus was cleaned between animals with 70% ethanol p.a. to avoid olfactory cues.

### Radial arm water maze

Radial arm water maze (RAWM) was performed following a published protocol [89] with minor changes using an in house built setup based on the authors’ specifications containing six arms extending out of a central area. The setup was built out of white plastic and positioned in white painted water so that the walls protruded 20 cm above the water surface. Briefly, mice were trained in RAWM to search for a platform submerged 1 cm below the water surface at the end of the goal arm. On day 1, animals were given two trials to learn to escape from water on a platform tagged with a flag (visible platform task) starting from alternating arms. Next, the flag was removed, visual cues (contrast-reach graphical forms like a cross, concentric circles, stripes etc.) were fixed on terminal walls of arms and mice were trained during 4 trials to find a platform submerged under water surface (hidden platform task). The duration of each trial (both in visible and hidden task) was 90 s; during this time mice were allowed to swim to the goal arm guided by visual cues. In case of an error defined as choice of a different arm than the goal arm, mice were immediately removed from the wrong arm, put gently again into the start arm and released. Entries into a goal arm, even if the platform was not located, were not counted as errors. If animals entered the wrong arm and after being brought to the start position repeated again the same wrong choice, this was counted as a “working error”. The procedure was repeated until mice found a platform (cut off time of 90 s). If mice were not able to find a platform within the given latency, they were gently guided to the proper position and were allowed to stay on the platform for 20 s for information acquisition. Intermittent to all trials, the water was gently mixed to avoid olfactory cues by urination or defecation. Mice were kept on the warm platform (37°C) to avoid body hypothermia between trials.

### Sucrose preference test

A sucrose preference test was set up in a standard plastic cage (Makrolon Type II) with normal bedding and with food *ad libitum*. Prior the experiment, two weight balanced water bottles were placed on each cage and weighted 24 hours later to exclude side preferences. On the second day, each cage was equipped with one bottle filled with 4% sucrose solution and one with water prepared freshly every day and provided at the same time point. Position of bottles (left versus right) was changed daily. Liquid intake was measured by weight of consumed water/sucrose solution over 24 h for 4 days.

### Drugs

Clozapine was purchased by Sigma-Aldrich (Germany) and dissolved in a drop of 0.1 M HCl, mixed with saline, pH adjusted to 5.3. Mice were injected i.p. with Clozapine (3 mg/kg) or with vehicle (saline pH 5.3) in volume of 10 ml/kg 30 min prior to behavioral testing.

### Statistical analysis

EEG and behavioral data are presented as means ± standard error of the mean (SEM) and were compared by ANOVA with Bonferroni (Bonf) post-hoc tests for repeated measurements or Mann-Whitney (MW) tests for genotype comparisons using GraphPad Prism 4 and 5 (GraphPad Software, San Diego, California). p values were indicated as p_Bonf_ or p_MW_, respectively. Moderate T-statistics and ANOVA were applied for gene expression data (using the corresponding R-packages at www.bioconductor.org and Genomics Suite, Partek). For qRT-PCR analysis the Mann-Whitney test was applied when normality testing failed and the unpaired two-tailed T-test for data showing normal distribution using GraphPad Prism 5. A p-values less than 0.05 were considered significant for all tests applied. Abbreviations for 2way ANOVA in figure legends are as follows: E, effect; I, interaction of factors.

## Supporting Information

Figure S1 **EEG recordings upon sleep deprivation.** A–C) Time course of the vigilance states wakefulness (A), NREM (B) and REM sleep (C) after 6 h of sleep deprivation (SD) performed from ZT0-6. Curves connect 2-h bin mean values (±SEM) expressed as percentage of recording time (I_genotype×time of day_: wakefulness F_(2, 20)_ = 0.23, p = 0.51; NREM F_(2,20)_ = 0.41, p = 0.67; REM F_(2,20)_ = 0.69, p = 0.51). WT, n = 7, filled circles. S1/2^-/-^, n = 8, empty circles. Data were analyzed with 2-way ANOVA. *: =  p<0.05 in two-tailed post hoc T-test). I, interaction of factors.
(TIF)

Figure S2 **Delta and theta wave oscillations of undisturbed sleep and upon sleep deprivation.** A) Baseline slow-wave activity and SD induced rebound sleep in S1/2^-/-^ mice. Slow-wave activity (SWA) was plotted over a 24 h period as percentage of the individual mean SWA over the last 900 NREM epochs in the baseline light period. Using 2-way ANOVA with the factors genotype and time, we detected no significant differences between baseline and SD recordings between WT and S1/2^-/-^ mice. However, a trend towards a higher SWA in S1/2^-/-^ mice was detected. WT: n = 7, S1/2^-/-^: n = 8. B) Group means (±SEM, WT: black bars; S1/2^-/-^: white bars) for mean theta peak frequency (TPF) during REM sleep in consecutive 6-h blocks (L1 = ZT0-6; L2 = ZT7-12, D1 = ZT13-18, D2 = ZT17-24) of baseline recordings (blank bars) and after 6-h SD (hatched bars). TPF varied with time-of-day and was significantly higher in the S1/2^-/-^ group during the 6 h following SD (2-way ANOVA: E_genotype_ F_(3,66)_ = 2.99 p = 0.04; E_time_ F_(2,66)_ = 4.61; p = 0.01; I_genotype×time_: F_(6, 66)_ = 0.46, p = 0.83; asterisks indicate significances between genotypes in post hoc T-test, **  =  p<0.01) WT: n = 7, S1/2^-/-^: n = 8. C–D) Power distribution in the 5–10 Hz range comparing fast-fourier transformed (FFT) EEG spectra of WT and S1/2^-/-^ during baseline conditions (C; I_frequency×genotype_ F_(12,117)_ = 0.57, p = 0.8588) and after SD (D; I_frequency×genotype_ F_(12,117)_ = 2.57, p = 0.0047). Note the significant shift of the theta component particularly between 6 and 7 Hz (p<0.05, post-hoc T-test). Data were analyzed with 2-way ANOVA. WT: n = 7, S1/2^-/-^: n = 8. SD, sleep deprivation; base, baseline. E, effect; I, interaction of factors.
(TIF)

Figure S3 **Divergent gene expression differences at ZT16 in the cortex of WT and S1/2^-/-^ mice as revealed by gene set enrichment analysis (GSEA).** A) P-value versus enrichment plot comparing cortical gene expression of WT with S1/2^-/-^ mice using the GENMAPP gene data sets. With a false-discovery rate (FDR) q-value cut-off set at 0.25 (default of the GSEA algorithm), six gene sets were found to be upregulated in WT samples whereas only two were significantly upregulated in S1/2^-/-^ mice (labeled by a black ellipses). The normalized enrichment score (NES) is plotted versus the FDR q-value (red dots) and the nominal p-value (black dots). B) Statistical parameters of the most significantly deregulated gene sets (FDR q-value <0.25). The most significantly WT versus S1/2^-/-^ upregulated gene sets at ZT16 correspond to molecular machineries involved in protein/RNA synthesis and turnover as well as oxidative phosphorylation, likely reflecting the higher metabolic demand in WT animals at ZT16 due to increased activity and wakefulness. In S1/2^-/-^ cortex, only two (highly similar) gene sets comprising class A and peptide GPCRs were detected as upregulated. SIZE, number of genes; ES, Enrichment score; NES, normalized enrichment score; Nom p-val, nominal p-value; FDR q-val, false discovery rate corrected q-value; FWER p-val, family wise error rate. C-D) Enrichment plots of the top deregulated gene sets encoding for components of proteasome (gene rank order depicted as vertical lines left-shifted  =  up in WT) and members of the class A GPCR family (gene rank order depicted as vertical lines right-shifted  =  up in S1/2^-/-^). n = 2 per timepoint and genotype.
(TIF)

Figure S4 **Altered gene expression profiles of control, circadian and activity-regulated genes in the cortex of WT and S1/2^-/-^ mice under baseline and sleep deprivation conditions.** A) Schematic drawing of the experimental schedule. WT and S1/2^-/-^ controls (base) and WT and S1/2^-/-^ animals that were sleep deprived from ZT0-6 (SD) were sacrificed at ZT10-12 for cortex preparations and marker gene expression analysis (n = 4 per each condition and genotype). B) Relative gene expression changes of control (*Atp5b*, *Actb*), selected circadian (*Per1*, *Per2*) and immediate early gene products (*Fos*, *Egr1*) in cortex samples plotted as fold changes between baseline and SD values (base/SD) for WT and S1/2^-/-^ groups.
(TIF)

Figure S5 **S1/2^-/-^ mice show normal behavior in elevated plus maze, light-dark preference and tail suspension test.** A) S1/2^-/-^ mice display normal performance spending similar time in closed arms in elevated plus maze when compared with WT controls (p_MW_ = 0.8693). WT: n = 23, S1/2^-/-^: n = 21. B) Time spent in the dark compartment during light-dark preference test was similar between both genotypes (p_MW_ = 0.1917). WT: n = 25, S1/2^-/-^: n = 18. C) Tail suspension test did not found significant difference in struggling behavior in S1/2^-/-^ mice (p_MW_ = 0.9456). WT: n = 24, S1/2^-/-^: n = 21. D) S1/2^-/-^ mice consume similar volume of sucrose solution as WT controls (E_genotype_ F_(1,41)_ = 1.93; p = 0.1719). WT: n = 23, S1/2^-/-^: n = 20. wt: black bars. S1/2^-/-^: white bars. Data were analyzed with 2-way ANOVA or Mann-Whitney test (p_MW_) for pairwise comparisons. E, effect.
(TIF)

Figure S6 **Multiple testing has no significant effects on prepulse inhibition (PPI) in control C57Bl/6 wild type mice.** A) C57Bl/6 wild type mice (n = 11) were tested in PPI test on three consecutive days. There are no significant effects of multiple testing on PPI observed (E_time_ F_(2,60)_ = 1.93; p = 0.1539). B) Startle response was similar on three testing days (p = 0.9724). Data were analyzed with 2-way ANOVA (A) and 1-way ANOVA (B). E, effect.
(TIF)

Figure S7 **SHARP1/2 mutant and control mice show similar habituation to 120 dB pulse.** A) Naïve (not injected) S1/2^-/-^ mice and their wildtype littermates showed comparable habituation (E_time_ F_(1,44)_ = 21.92; p<0.0001) which was similar between the genotypes (E_genotype_ F_(1,44)_ = 0.52; p = 0.4766). WT: n = 25; S1/2^-/-^: n = 21. B) Mice injected with vehicle display habituation (E_time_ F_(1,44)_ = 13.73; p = 0.0006) which is not altered in mutants (E_genotype_ F_(1,44)_ = 0.15; p = 0.6974). WT: n = 25; S1/2^-/-^: n = 21. C) Animals treated with clozapine (3 mg/kg) habituate to startling pulse (E_time_ F_(1,38)_ = 5.93; p = 0.0197) independent of the genotype (E_genotype_ F_(1,38)_ = 0.28; p = 0.6008). WT: n = 20; S1/2^-/-^: n = 20. Data were analyzed with 2-way ANOVA. E, effect.
(TIF)

Figure S8 **S1/2^-/-^ mice respond stronger to clozapine treatment in the open field than WT controls.** A) In a familiar open field box, hyperactivity in vehicle injected S1/2^-/-^ mice was not evident (p_MW_ = 0.2380). Clozapine reduced distance travelled (E_treatment_ F_(1,38)_ = 103.89; p<0.0001). A 2-way ANOVA yielded a significant I_genotype×treatment_ (F_(1,38)_ = 5.11; p = 0.0296). B) Vehicle treated S1/2^-/-^ mice showed tendency to spend more time in the center of the familiar test arena (p_MW_ = 0.2250). Time spent in the center of the open field was reduced by clozapine (F_(1,38)_ = 13.10; p = 0.0009) in both genotypes. However, clozapine effects were stronger in S1/2^-/-^ mice (I_genotype×treatment_ F_(1,38)_ = 3.57; p = 0.0665). Vehicle treated mice: WT: n = 25; S1/2^-/-^: n = 21; clozapine: WT: n = 20; S1/2^-/-^: n = 20.E, effect; I, interaction of factors.
(TIF)

Figure S9 **Description of network objects.** Graphical symbols describing functional classification of network objects (nodes  =  genes or functionally grouped genes; edges  =  connections between nodes) represent default settings by the MetaCore software as depicted.
(TIF)

Table S1 **Genes differentially regulated at ZT4 versus ZT16 in cortex samples of WT and S1/2^-/-^ mice.** The selection cut-off was set to fold-change (FC) of at least 1.5 and p-value of <0.05 in WT (including all genes/probe sets with yellow and blue background). Note, that *Penk* was detected with two probe sets to be upregulated at ZT16 in the WT cortex and that only one gene (*Chl1*) showed a significant downregulation at ZT16 (indicated by a negative FC). In S1/2^-/-^ mice, five genes were detected to be significantly de-regulated between ZT4 and ZT16 with a p-value <0.05 (blue background), the corresponding FC values were, however, reduced compared to the WT. (n = 4 independent samples per genotype and two replicates per timepoint, p<0.05 was considered significant by ANOVA). We validated the attenuated cortical gene expression in S1/2^-/-^ mice with quantitative RT-PCR (qRT-PCR) for 10 genes in LD and DD (indicated at the table on the right: ZT4 vs 16 and CT4 vs 16, correspondingly (n = 3 per timepoint per genotype).
(TIF)

Table S2 **Normalized expression values of microarray data.** Depicted are the means and corresponding standard deviation (SD) of normalized microarray data from ZT4 and ZT16 cortex samples of WT and S1/2^-/-^ mice (n = 2 per timepoint per genotype).
(TIF)

Table S3 **List of protein names, gene symbols and synonyms.**
(TIF)

Table S4 **Table of genes and primer sequences used for gene expression analysis.**
(TIF)
