# Supplementary figures and images for: Mice Lacking the Circadian Modulators SHARP1 and SHARP2 Display Altered Sleep and Mixed State Endophenotypes of Psychiatric Disorders
Source: PLoS One. 2014 Oct 23;9(10):e110310. doi: 10.1371/journal.pone.0110310 (PMC4207740; doi:10.1371/journal.pone.0110310)

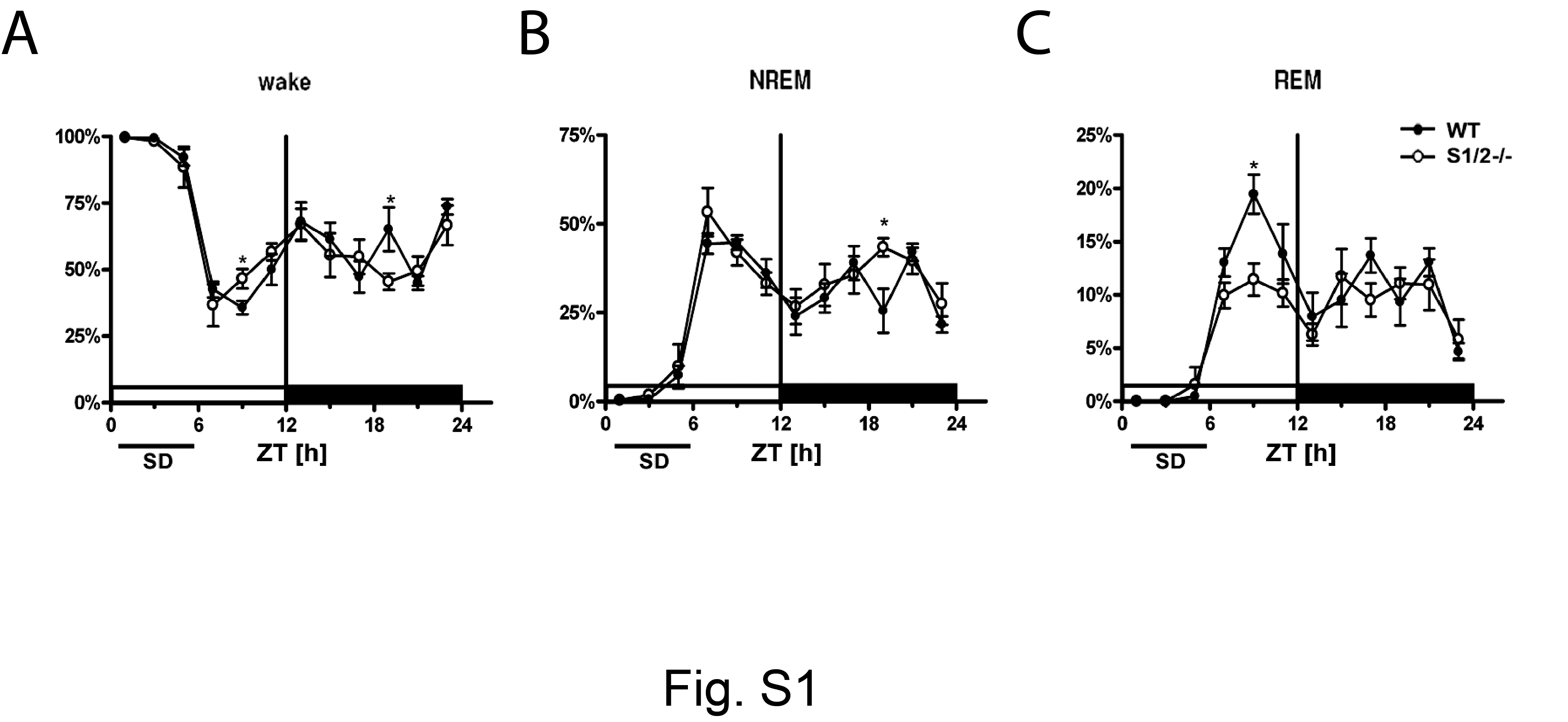

Supplement: Figure S1 — EEG recordings upon sleep deprivation. A–C) Time course of the vigilance states wakefulness (A), NREM (B) and REM sleep (C) after 6 h of sleep deprivation (SD) performed from ZT0-6. Curves connect 2-h bin mean values (±SEM) expressed as percentage of recording time (Igenotype×time of day: wakefulness F(2, 20) = 0.23, p = 0.51; NREM F(2,20) = 0.41, p = 0.67; REM F(2,20) = 0.69, p = 0.51). WT, n = 7, filled circles. S1/2-/-, n = 8, empty circles. Data were analyzed with 2-way ANOVA. *: = p<0.05 in two-tailed post hoc T-test). I, interaction of factors. (TIF) [file pone.0110310.s001.tif]

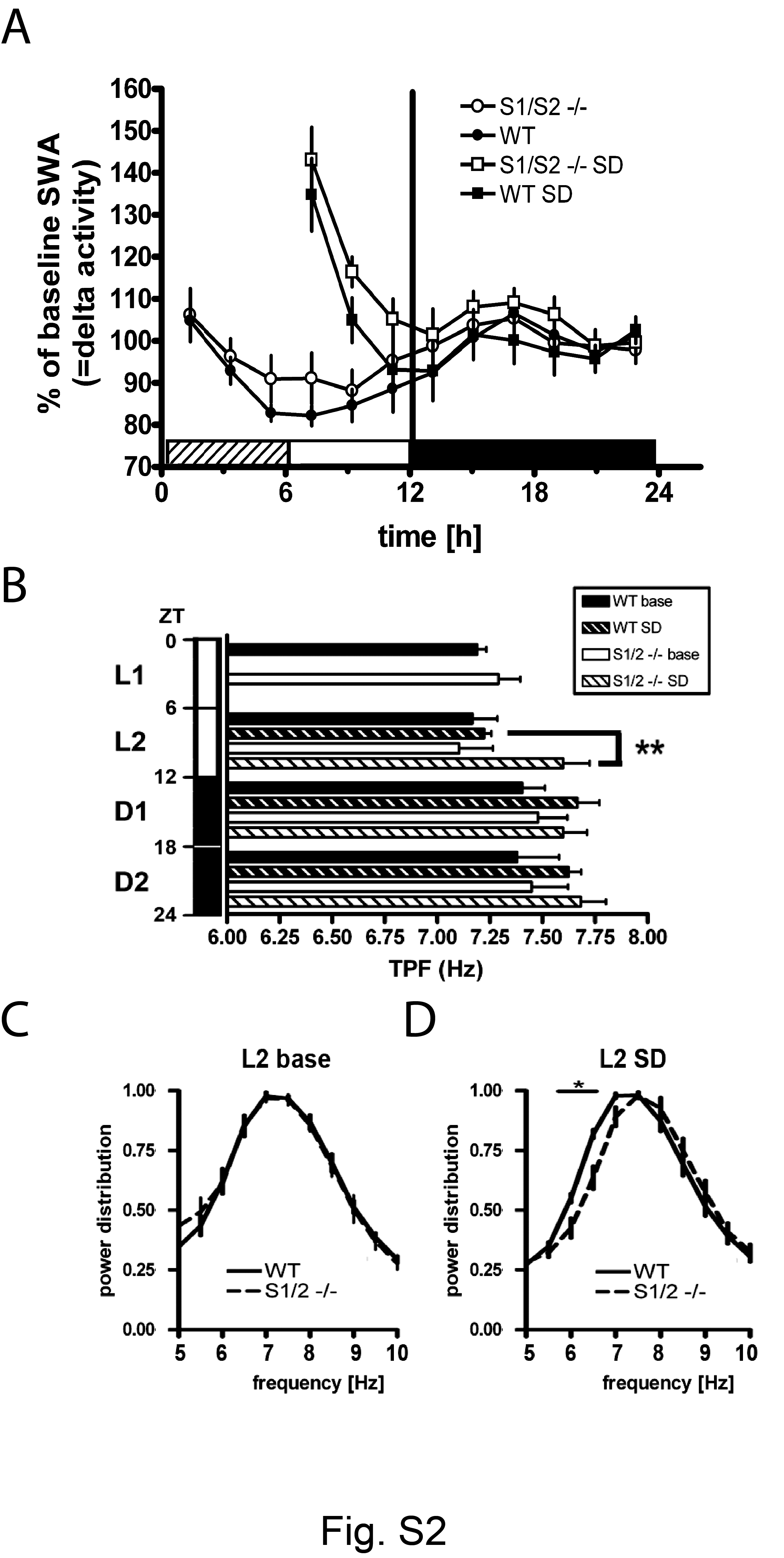

Supplement: Figure S2 — Delta and theta wave oscillations of undisturbed sleep and upon sleep deprivation. A) Baseline slow-wave activity and SD induced rebound sleep in S1/2-/- mice. Slow-wave activity (SWA) was plotted over a 24 h period as percentage of the individual mean SWA over the last 900 NREM epochs in the baseline light period. Using 2-way ANOVA with the factors genotype and time, we detected no significant differences between baseline and SD recordings between WT and S1/2-/- mice. However, a trend towards a higher SWA in S1/2-/- mice was detected. WT: n = 7, S1/2-/-: n = 8. B) Group means (±SEM, WT: black bars; S1/2-/-: white bars) for mean theta peak frequency (TPF) during REM sleep in consecutive 6-h blocks (L1 = ZT0-6; L2 = ZT7-12, D1 = ZT13-18, D2 = ZT17-24) of baseline recordings (blank bars) and after 6-h SD (hatched bars). TPF varied with time-of-day and was significantly higher in the S1/2-/- group during the 6 h following SD (2-way ANOVA: Egenotype F(3,66) = 2.99 p = 0.04; Etime F(2,66) = 4.61; p = 0.01; Igenotype×time: F(6, 66) = 0.46, p = 0.83; asterisks indicate significances between genotypes in post hoc T-test, ** = p<0.01) WT: n = 7, S1/2-/-: n = 8. C–D) Power distribution in the 5–10 Hz range comparing fast-fourier transformed (FFT) EEG spectra of WT and S1/2-/- during baseline conditions (C; Ifrequency×genotype F(12,117) = 0.57, p = 0.8588) and after SD (D; Ifrequency×genotype F(12,117) = 2.57, p = 0.0047). Note the significant shift of the theta component particularly between 6 and 7 Hz (p<0.05, post-hoc T-test). Data were analyzed with 2-way ANOVA. WT: n = 7, S1/2-/-: n = 8. SD, sleep deprivation; base, baseline. E, effect; I, interaction of factors. (TIF) [file pone.0110310.s002.tif]

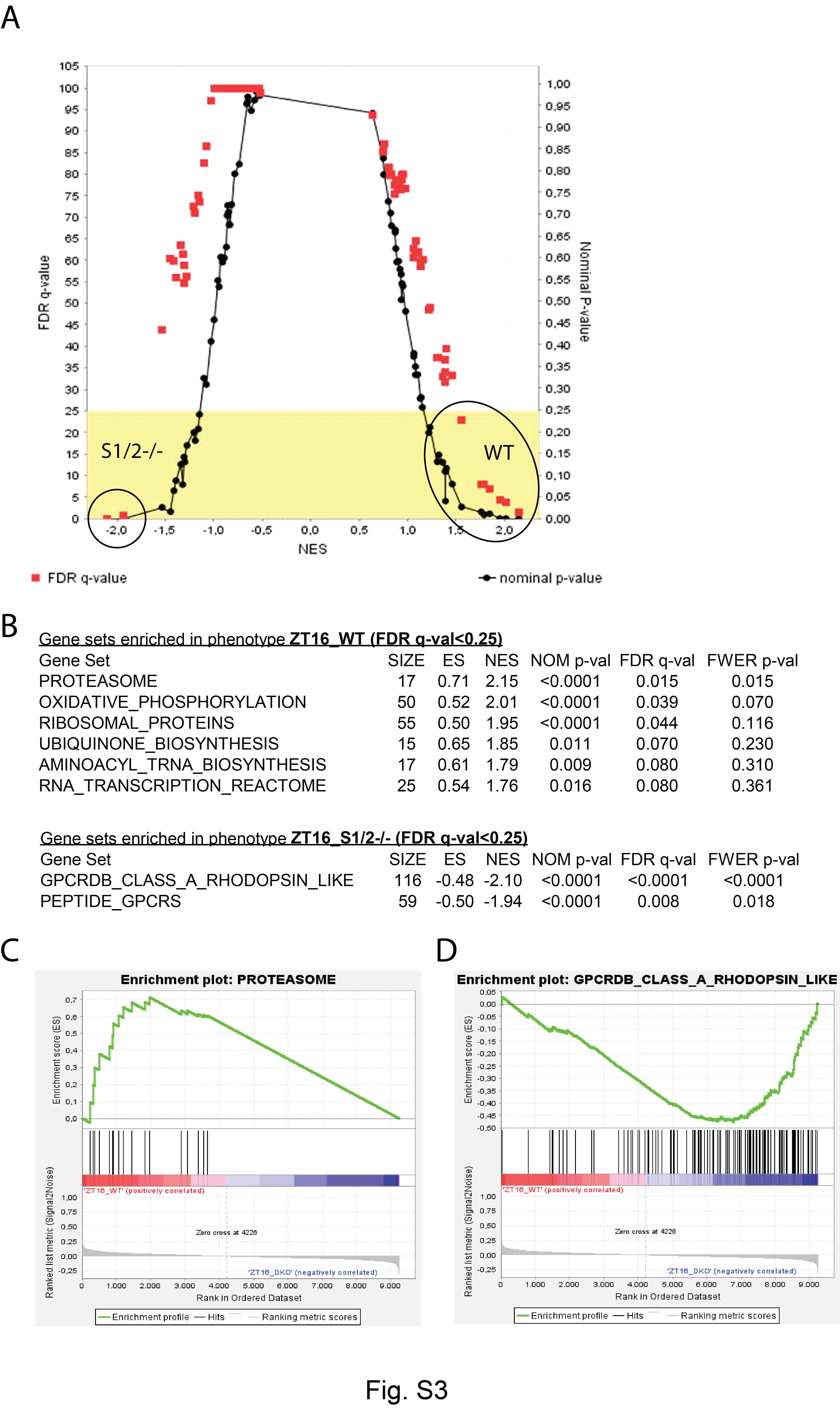

Supplement: Figure S3 — Divergent gene expression differences at ZT16 in the cortex of WT and S1/2-/- mice as revealed by gene set enrichment analysis (GSEA). A) P-value versus enrichment plot comparing cortical gene expression of WT with S1/2-/- mice using the GENMAPP gene data sets. With a false-discovery rate (FDR) q-value cut-off set at 0.25 (default of the GSEA algorithm), six gene sets were found to be upregulated in WT samples whereas only two were significantly upregulated in S1/2-/- mice (labeled by a black ellipses). The normalized enrichment score (NES) is plotted versus the FDR q-value (red dots) and the nominal p-value (black dots). B) Statistical parameters of the most significantly deregulated gene sets (FDR q-value <0.25). The most significantly WT versus S1/2-/- upregulated gene sets at ZT16 correspond to molecular machineries involved in protein/RNA synthesis and turnover as well as oxidative phosphorylation, likely reflecting the higher metabolic demand in WT animals at ZT16 due to increased activity and wakefulness. In S1/2-/- cortex, only two (highly similar) gene sets comprising class A and peptide GPCRs were detected as upregulated. SIZE, number of genes; ES, Enrichment score; NES, normalized enrichment score; Nom p-val, nominal p-value; FDR q-val, false discovery rate corrected q-value; FWER p-val, family wise error rate. C-D) Enrichment plots of the top deregulated gene sets encoding for components of proteasome (gene rank order depicted as vertical lines left-shifted = up in WT) and members of the class A GPCR family (gene rank order depicted as vertical lines right-shifted = up in S1/2-/-). n = 2 per timepoint and genotype. (TIF) [file pone.0110310.s003.tif]

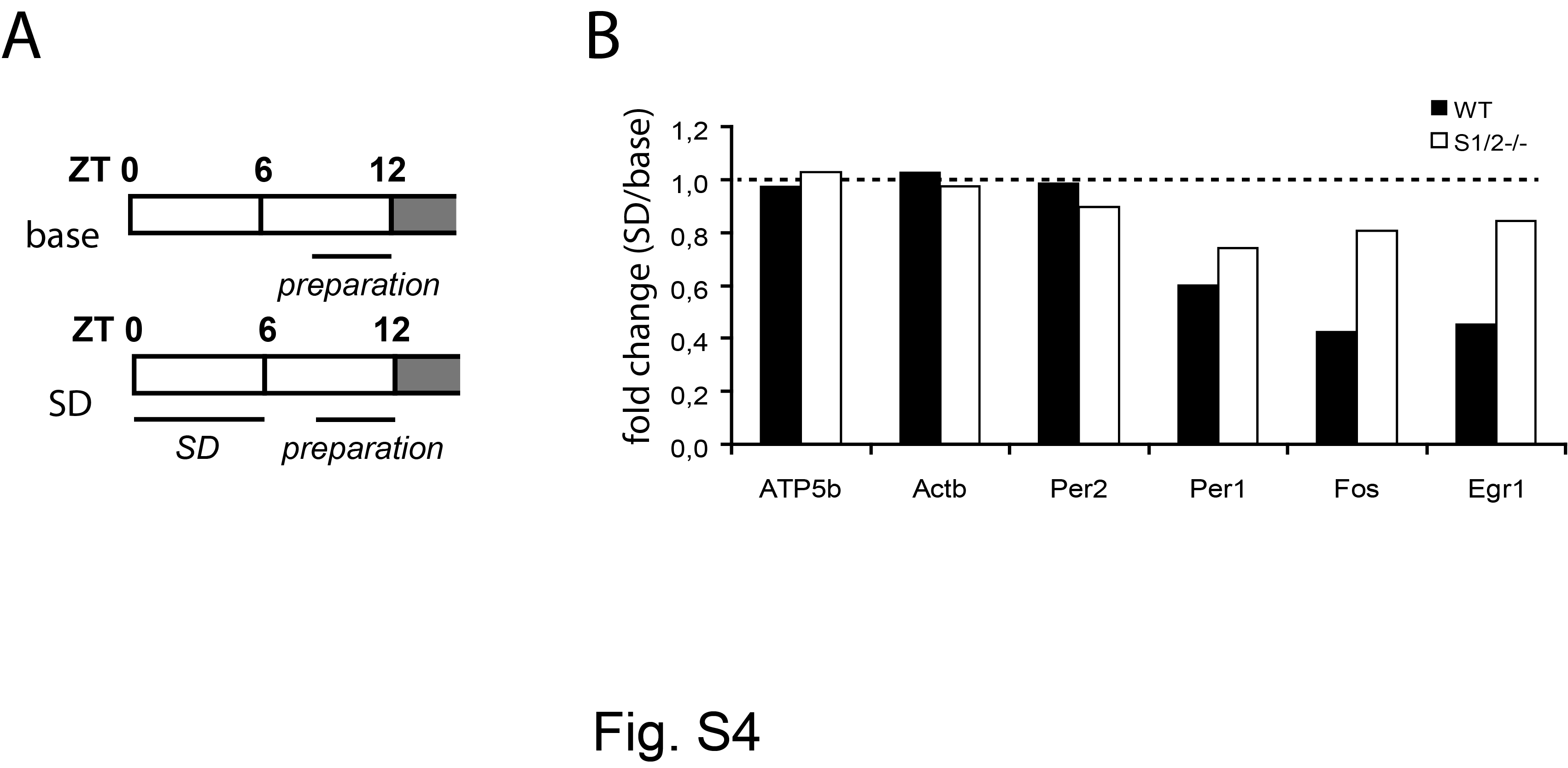

Supplement: Figure S4 — Altered gene expression profiles of control, circadian and activity-regulated genes in the cortex of WT and S1/2-/- mice under baseline and sleep deprivation conditions. A) Schematic drawing of the experimental schedule. WT and S1/2-/- controls (base) and WT and S1/2-/- animals that were sleep deprived from ZT0-6 (SD) were sacrificed at ZT10-12 for cortex preparations and marker gene expression analysis (n = 4 per each condition and genotype). B) Relative gene expression changes of control (Atp5b, Actb), selected circadian (Per1, Per2) and immediate early gene products (Fos, Egr1) in cortex samples plotted as fold changes between baseline and SD values (base/SD) for WT and S1/2-/- groups. (TIF) [file pone.0110310.s004.tif]

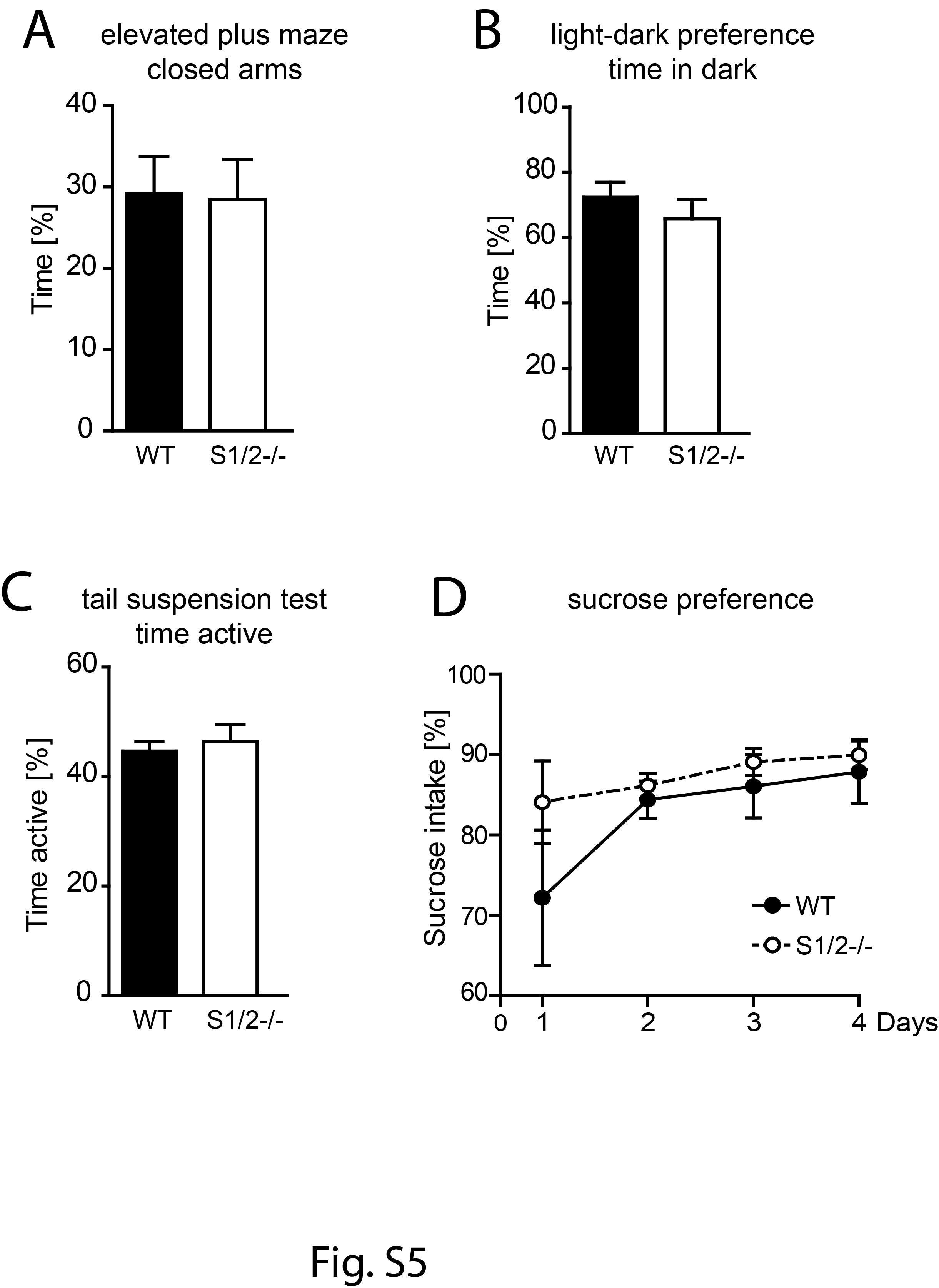

Supplement: Figure S5 — S1/2-/- mice show normal behavior in elevated plus maze, light-dark preference and tail suspension test. A) S1/2-/- mice display normal performance spending similar time in closed arms in elevated plus maze when compared with WT controls (pMW = 0.8693). WT: n = 23, S1/2-/-: n = 21. B) Time spent in the dark compartment during light-dark preference test was similar between both genotypes (pMW = 0.1917). WT: n = 25, S1/2-/-: n = 18. C) Tail suspension test did not found significant difference in struggling behavior in S1/2-/- mice (pMW = 0.9456). WT: n = 24, S1/2-/-: n = 21. D) S1/2-/- mice consume similar volume of sucrose solution as WT controls (Egenotype F(1,41) = 1.93; p = 0.1719). WT: n = 23, S1/2-/-: n = 20. wt: black bars. S1/2-/-: white bars. Data were analyzed with 2-way ANOVA or Mann-Whitney test (pMW) for pairwise comparisons. E, effect. (TIF) [file pone.0110310.s005.tif]

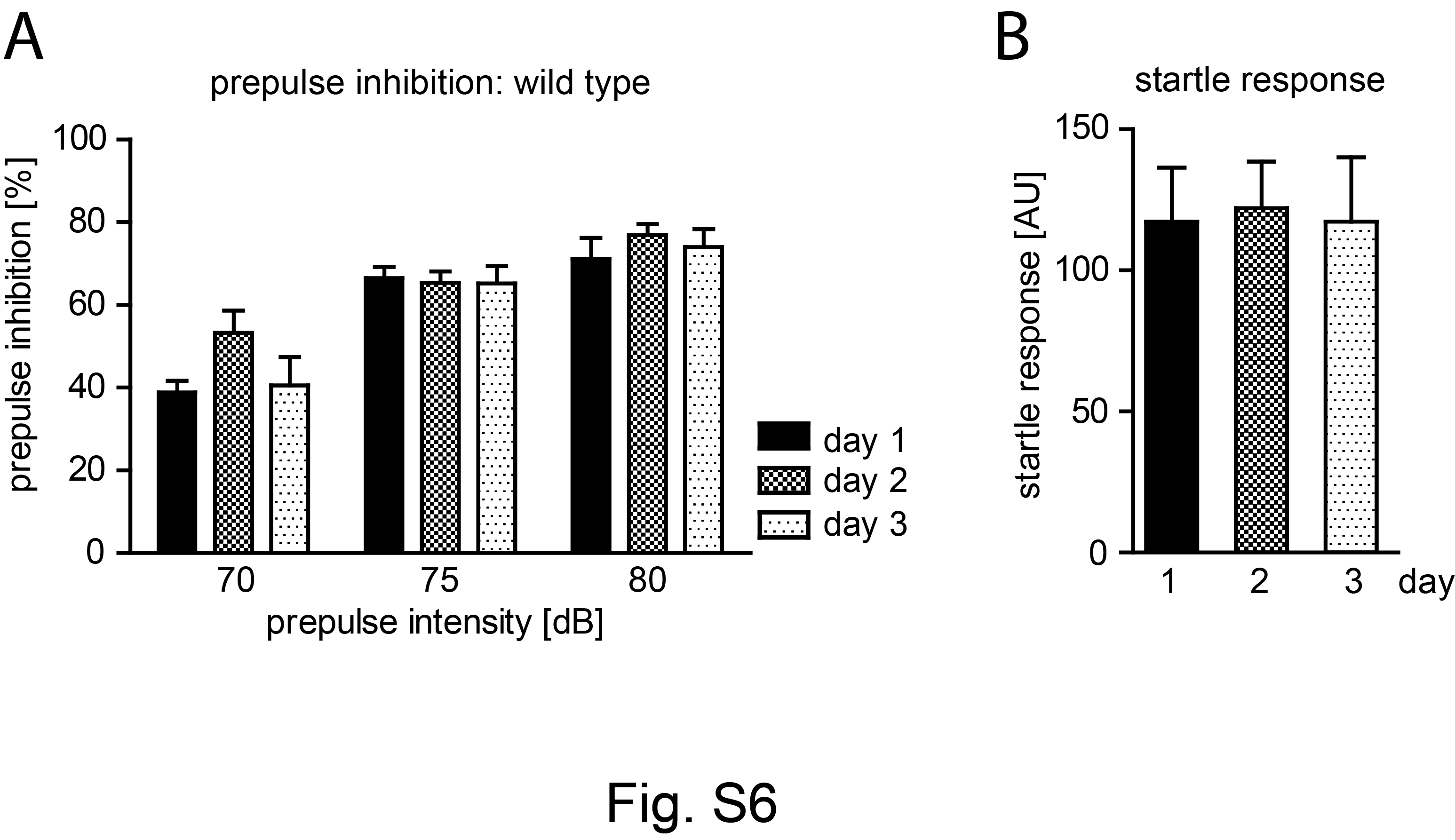

Supplement: Figure S6 — Multiple testing has no significant effects on prepulse inhibition (PPI) in control C57Bl/6 wild type mice. A) C57Bl/6 wild type mice (n = 11) were tested in PPI test on three consecutive days. There are no significant effects of multiple testing on PPI observed (Etime F(2,60) = 1.93; p = 0.1539). B) Startle response was similar on three testing days (p = 0.9724). Data were analyzed with 2-way ANOVA (A) and 1-way ANOVA (B). E, effect. (TIF) [file pone.0110310.s006.tif]

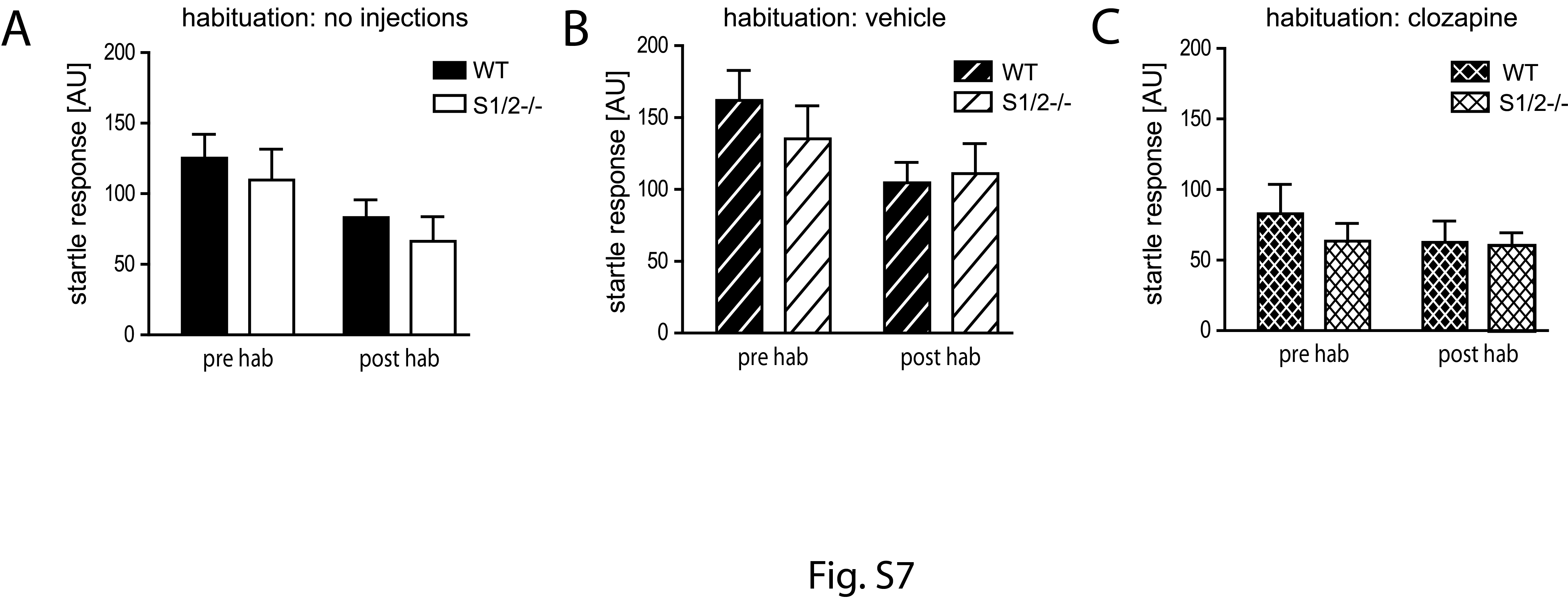

Supplement: Figure S7 — SHARP1/2 mutant and control mice show similar habituation to 120 dB pulse. A) Naïve (not injected) S1/2-/- mice and their wildtype littermates showed comparable habituation (Etime F(1,44) = 21.92; p<0.0001) which was similar between the genotypes (Egenotype F(1,44) = 0.52; p = 0.4766). WT: n = 25; S1/2-/-: n = 21. B) Mice injected with vehicle display habituation (Etime F(1,44) = 13.73; p = 0.0006) which is not altered in mutants (Egenotype F(1,44) = 0.15; p = 0.6974). WT: n = 25; S1/2-/-: n = 21. C) Animals treated with clozapine (3 mg/kg) habituate to startling pulse (Etime F(1,38) = 5.93; p = 0.0197) independent of the genotype (Egenotype F(1,38) = 0.28; p = 0.6008). WT: n = 20; S1/2-/-: n = 20. Data were analyzed with 2-way ANOVA. E, effect. (TIF) [file pone.0110310.s007.tif]

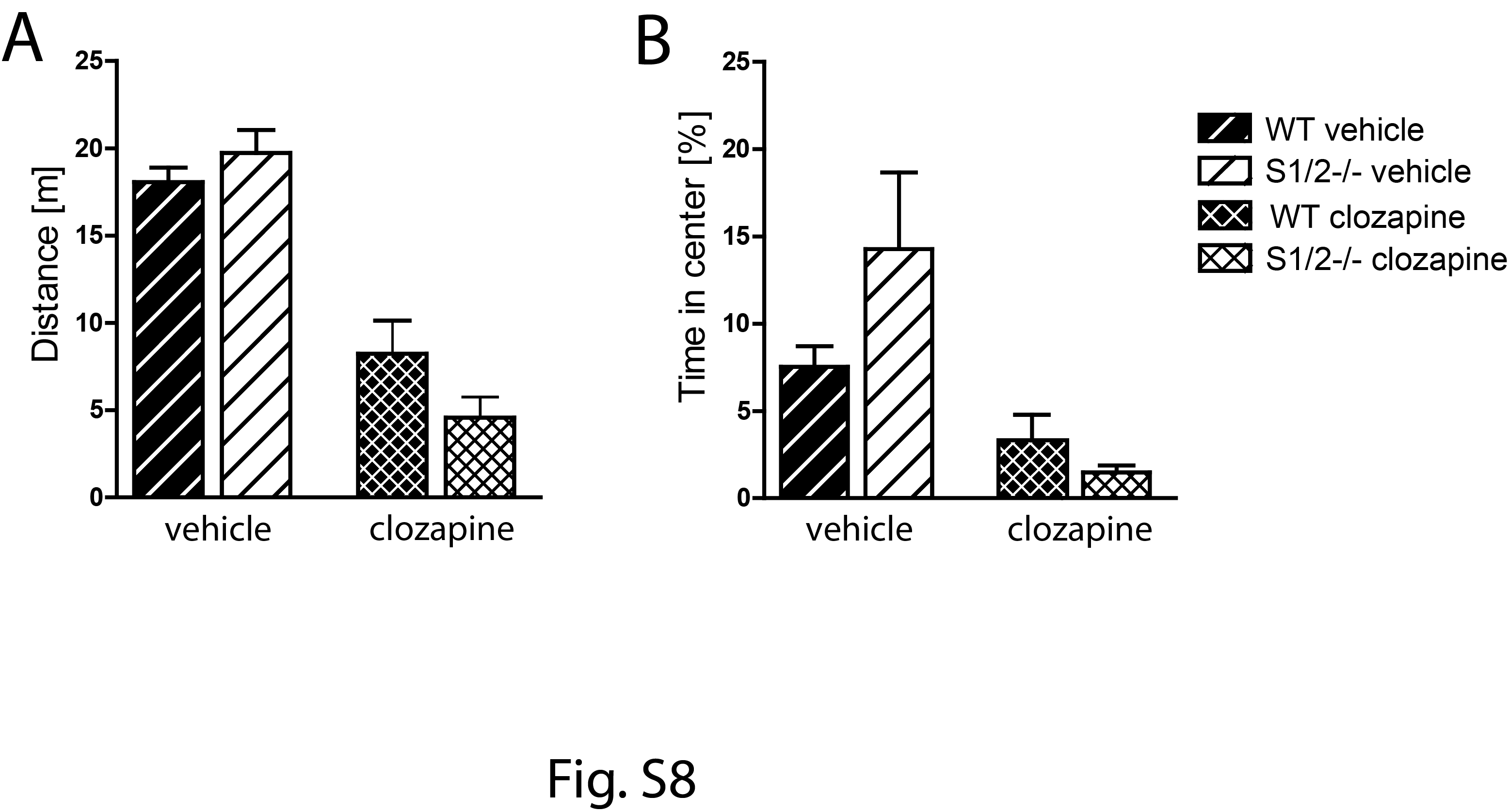

Supplement: Figure S8 — S1/2-/- mice respond stronger to clozapine treatment in the open field than WT controls. A) In a familiar open field box, hyperactivity in vehicle injected S1/2-/- mice was not evident (pMW = 0.2380). Clozapine reduced distance travelled (Etreatment F(1,38) = 103.89; p<0.0001). A 2-way ANOVA yielded a significant Igenotype×treatment (F(1,38) = 5.11; p = 0.0296). B) Vehicle treated S1/2-/- mice showed tendency to spend more time in the center of the familiar test arena (pMW = 0.2250). Time spent in the center of the open field was reduced by clozapine (F(1,38) = 13.10; p = 0.0009) in both genotypes. However, clozapine effects were stronger in S1/2-/- mice (Igenotype×treatment F(1,38) = 3.57; p = 0.0665). Vehicle treated mice: WT: n = 25; S1/2-/-: n = 21; clozapine: WT: n = 20; S1/2-/-: n = 20.E, effect; I, interaction of factors. (TIF) [file pone.0110310.s008.tif]

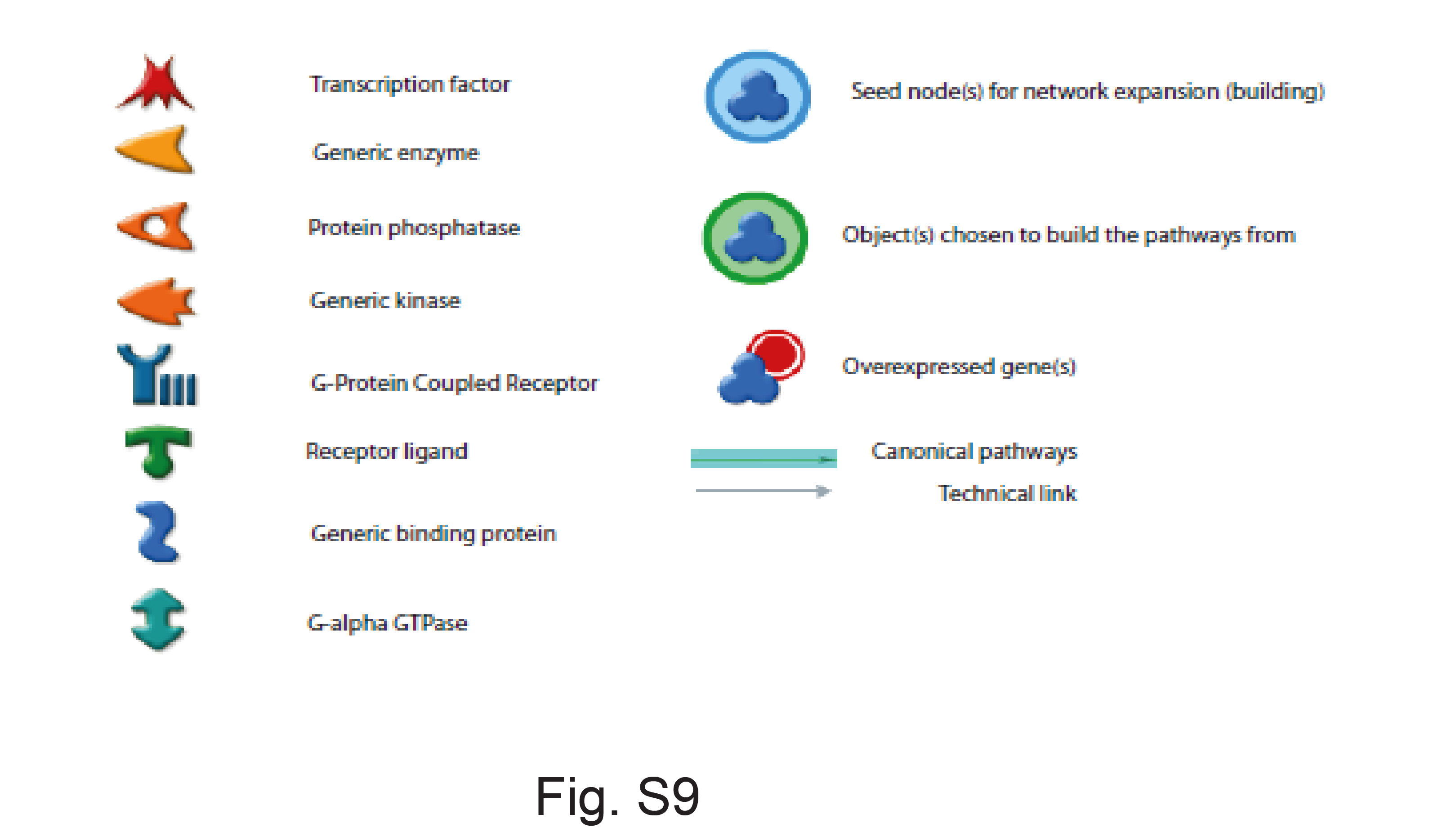

Supplement: Figure S9 — Description of network objects. Graphical symbols describing functional classification of network objects (nodes = genes or functionally grouped genes; edges = connections between nodes) represent default settings by the MetaCore software as depicted. (TIF) [file pone.0110310.s009.tif]

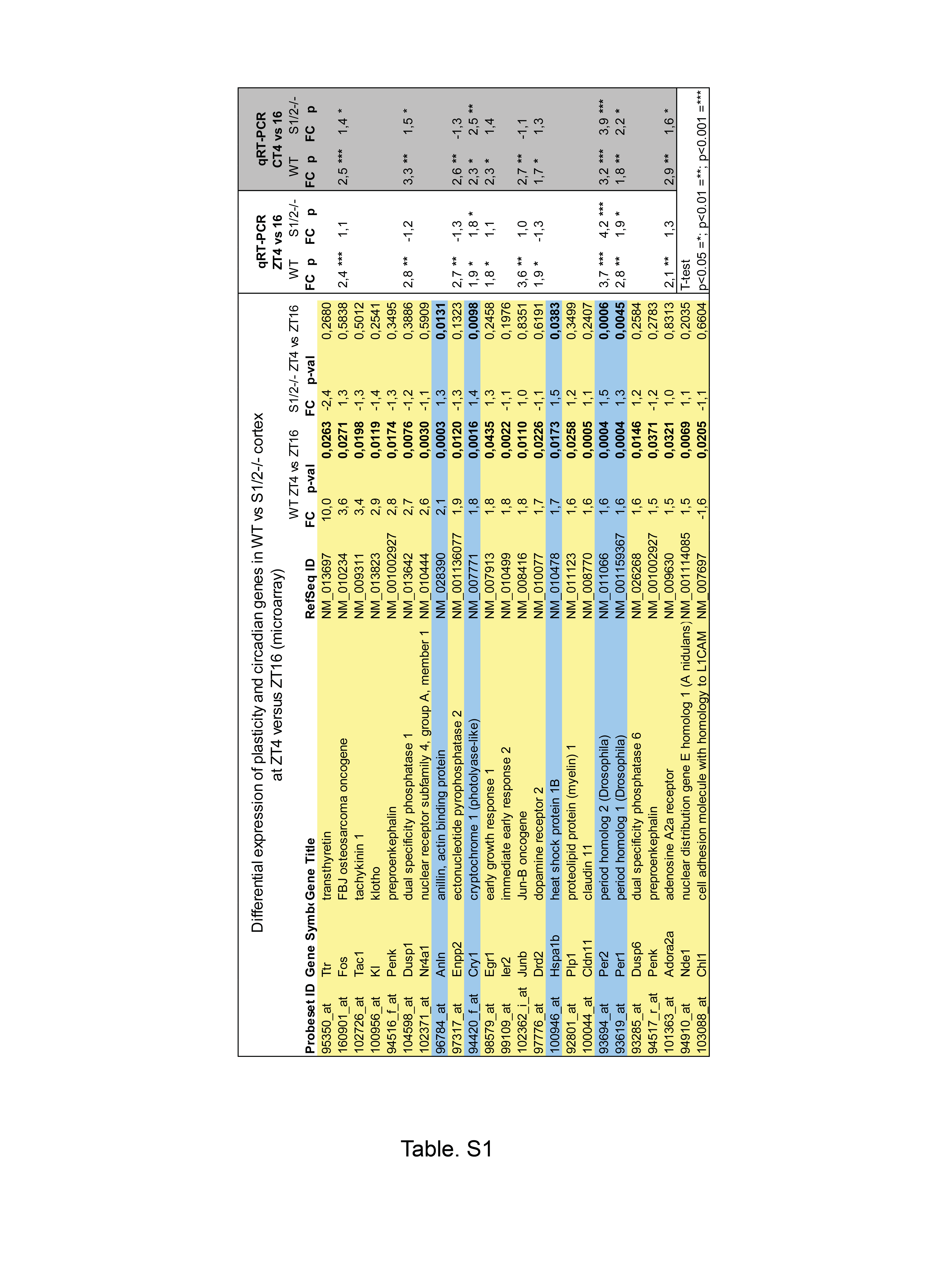

Supplement: Table S1 — Genes differentially regulated at ZT4 versus ZT16 in cortex samples of WT and S1/2-/- mice. The selection cut-off was set to fold-change (FC) of at least 1.5 and p-value of <0.05 in WT (including all genes/probe sets with yellow and blue background). Note, that Penk was detected with two probe sets to be upregulated at ZT16 in the WT cortex and that only one gene (Chl1) showed a significant downregulation at ZT16 (indicated by a negative FC). In S1/2-/- mice, five genes were detected to be significantly de-regulated between ZT4 and ZT16 with a p-value <0.05 (blue background), the corresponding FC values were, however, reduced compared to the WT. (n = 4 independent samples per genotype and two replicates per timepoint, p<0.05 was considered significant by ANOVA). We validated the attenuated cortical gene expression in S1/2-/- mice with quantitative RT-PCR (qRT-PCR) for 10 genes in LD and DD (indicated at the table on the right: ZT4 vs 16 and CT4 vs 16, correspondingly (n = 3 per timepoint per genotype). (TIF) [file pone.0110310.s010.tif]

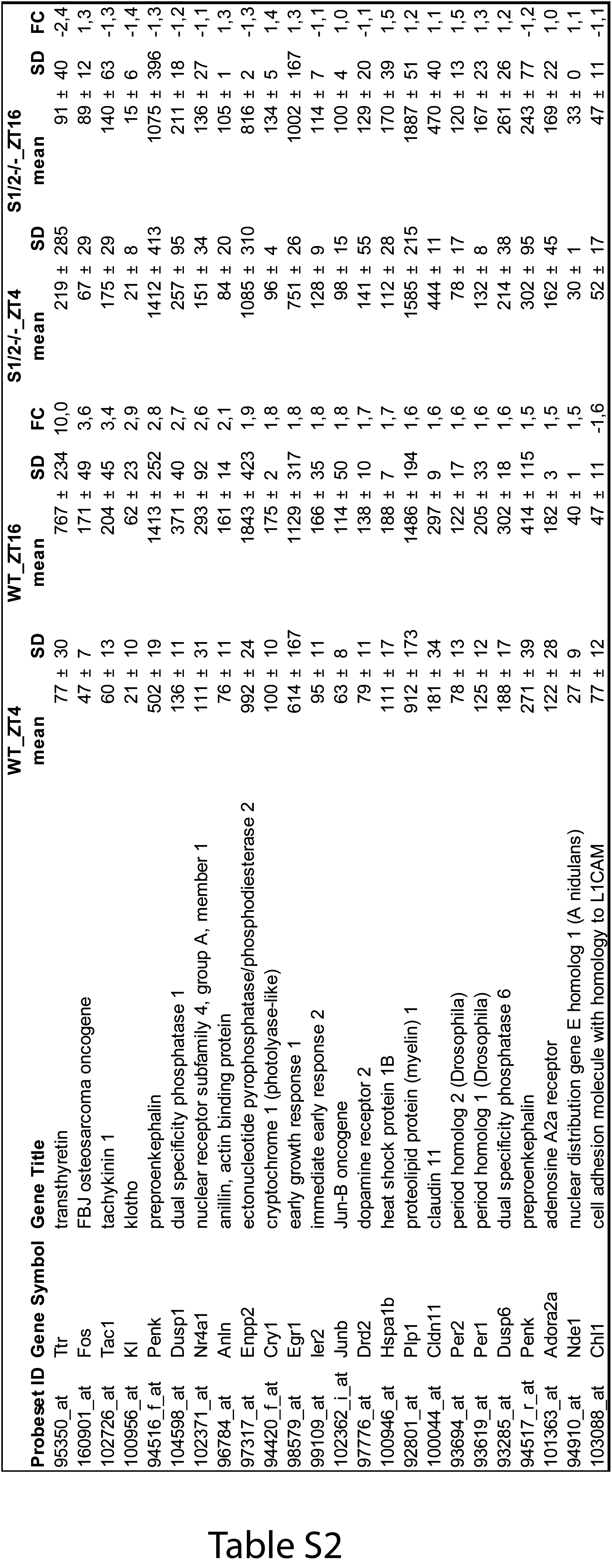

Supplement: Table S2 — Normalized expression values of microarray data. Depicted are the means and corresponding standard deviation (SD) of normalized microarray data from ZT4 and ZT16 cortex samples of WT and S1/2-/- mice (n = 2 per timepoint per genotype). (TIF) [file pone.0110310.s011.tif]

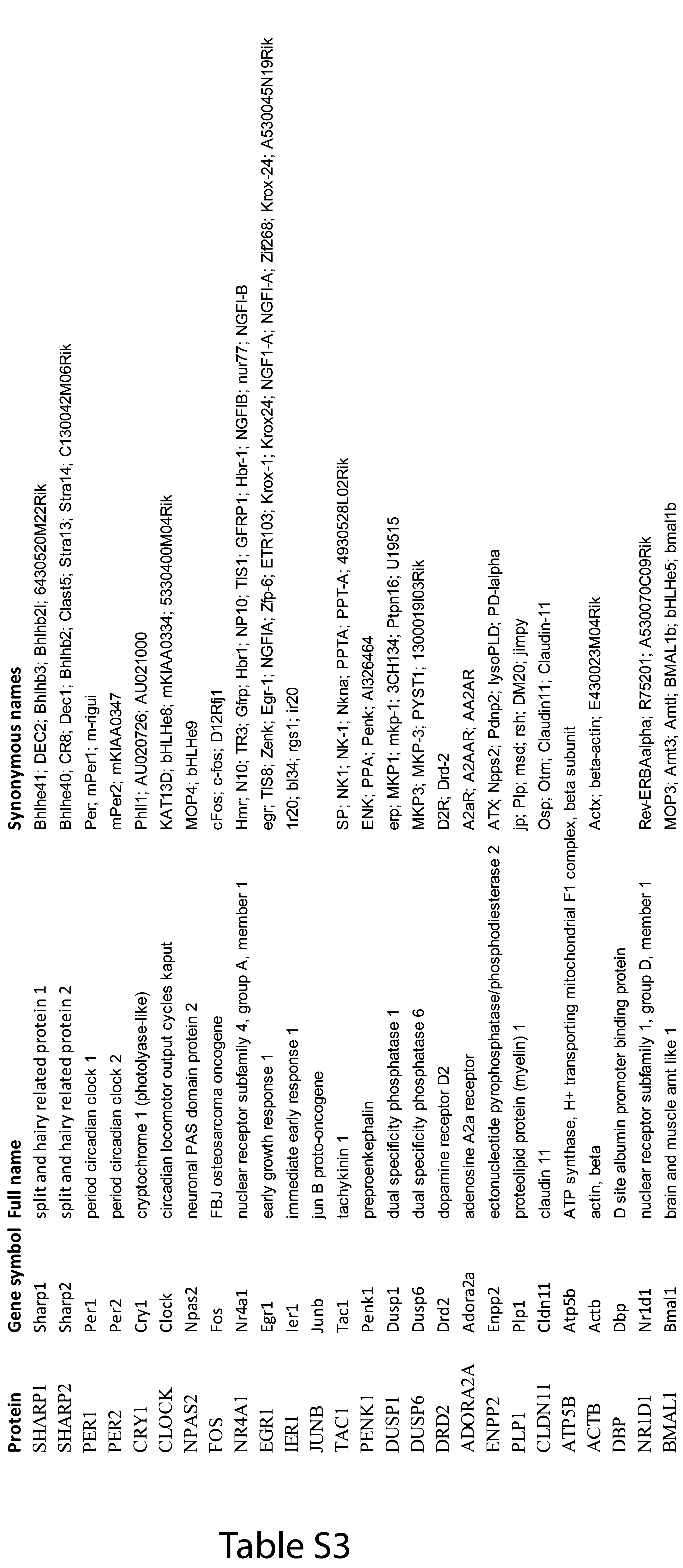

Supplement: Table S3 — List of protein names, gene symbols and synonyms. (TIF) [file pone.0110310.s012.tif]

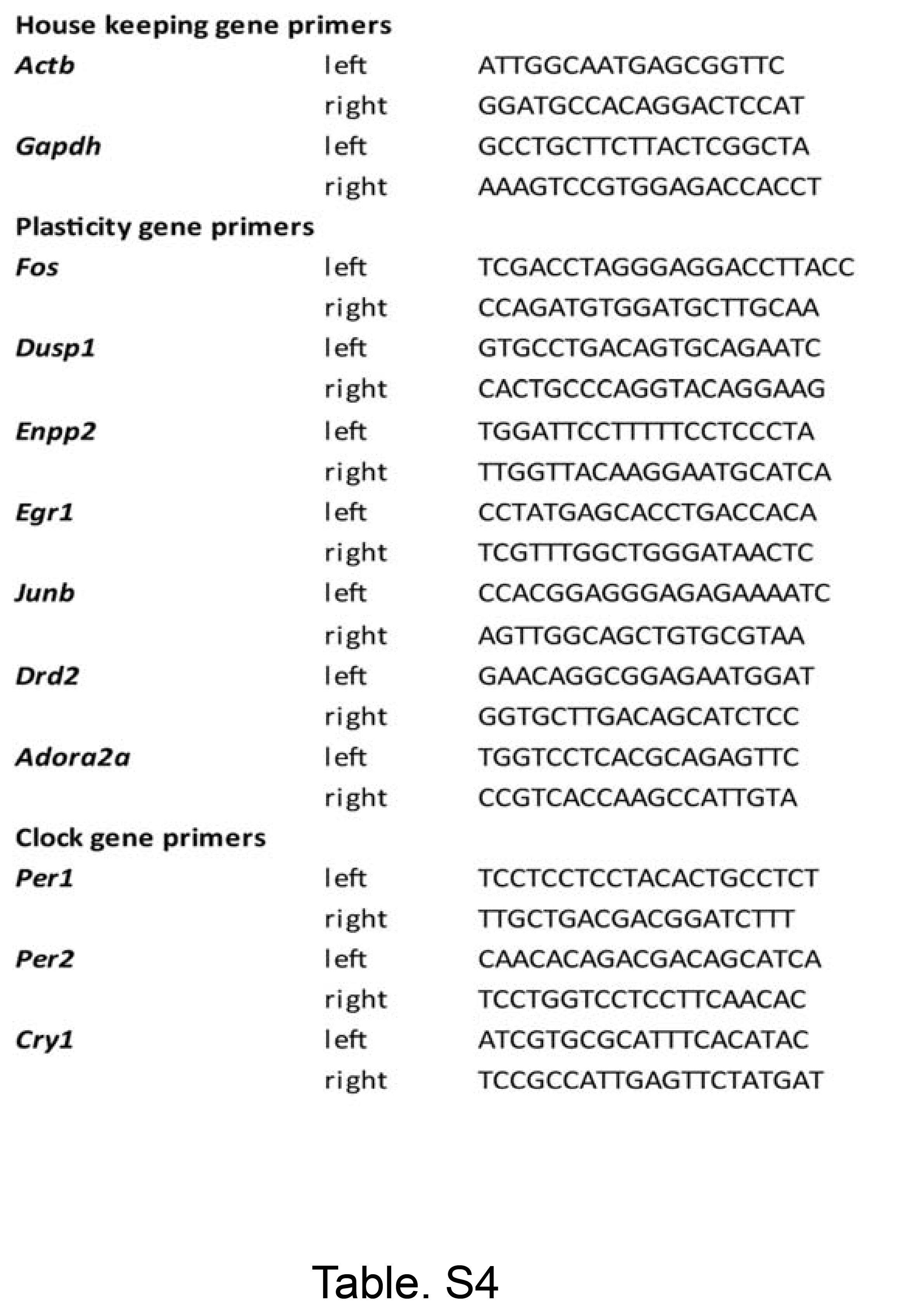

Supplement: Table S4 — Table of genes and primer sequences used for gene expression analysis. (TIF) [file pone.0110310.s013.tif]
